# Deficiency of the SMOC2 matricellular protein impairs bone healing and produces age-dependent bone loss

**DOI:** 10.1038/s41598-020-71749-6

**Published:** 2020-09-09

**Authors:** Supawich Morkmued, François Clauss, Brigitte Schuhbaur, Valérie Fraulob, Eric Mathieu, Joseph Hemmerlé, Hans Clevers, Bon-Kyoung Koo, Pascal Dollé, Agnès Bloch-Zupan, Karen Niederreither

**Affiliations:** 1 Developmental Biology and Stem Cells Department, Institute of Genetics and of Molecular and Cellular Biology (IGBMC), 1 rue Laurent Fries, BP 10142, 67404 Illkirch, France; 2 Centre National de la Recherche Scientifique, UMR7104, Illkirch, France; 3 Institut National de la Santé et de la Recherche Médicale, INSERM U1258, Illkirch, France; 4 Université de Strasbourg, Illkirch, France; 5 Faculty of Dentistry, Pediatrics Division, Preventive Department, Khon Kaen University, Khon Kaen, Thailand; 6 Faculté de Chirurgie Dentaire, Université de Strasbourg, 8 rue Ste Elisabeth, 67000 Strasbourg, France; 7 Hôpitaux Universitaires de Strasbourg, Pôle de Médecine et Chirurgie Bucco-Dentaires, Centre de Référence des Maladies Rares Orales et Dentaires, CRMR O Rares, Filière TETECOU, ERN CRANIO, 1 place de l’Hôpital, 67000 Strasbourg, France; 8 Regenerative NanoMedicine, INSERM UMR1260, FMTS, Hôpitaux Universitaires de Strasbourg, 11 rue Humann, 67000 Strasbourg, France; 9 Biomaterials and Bioengineering, Université de Strasbourg, INSERM UMR1121, 11 rue Humann, 67000 Strasbourg, France; 10 Hubrecht Institute, University Medical Center Utrecht, and University Utrecht, Utrecht, The Netherlands; 11 Faculté de Médecine, Université de Strasbourg, FMTS, 4 Rue Kirschleger, 67000 Strasbourg, France; 12 Eastman Dental Institute, University College London, London, UK

**Keywords:** Development, Dental diseases, Bone

## Abstract

Secreted extracellular matrix components which regulate craniofacial development could be reactivated and play roles in adult wound healing. We report a patient with a loss-of-function of the secreted matricellular protein SMOC2 (SPARC related modular calcium binding 2) presenting severe oligodontia, microdontia, tooth root deficiencies, alveolar bone hypoplasia, and a range of skeletal malformations. Turning to a mouse model, *Smoc2-GFP* reporter expression indicates SMOC2 dynamically marks a range of dental and bone progenitors. While germline *Smoc2* homozygous mutants are viable, tooth number anomalies, reduced tooth size, altered enamel prism patterning, and spontaneous age-induced periodontal bone and root loss are observed in this mouse model. Whole-genome RNA-sequencing analysis of embryonic day (E) 14.5 cap stage molars revealed reductions in early expressed enamel matrix components (*Odontogenic ameloblast-associated protein*) and dentin dysplasia targets (*Dentin matrix acidic phosphoprotein 1*). We tested if like other matricellular proteins SMOC2 was required for regenerative repair. We found that the *Smoc2-GFP* reporter was reactivated in adjacent periodontal tissues 4 days after tooth avulsion injury. Following maxillary tooth injury, *Smoc2*^*−/−*^ mutants had increased osteoclast activity and bone resorption surrounding the extracted molar. Interestingly, a 10-day treatment with the cyclooxygenase 2 (COX2) inhibitor ibuprofen (30 mg/kg body weight) blocked tooth injury-induced bone loss in *Smoc2*^*−/−*^ mutants, reducing *matrix metalloprotease (Mmp)9*. Collectively, our results indicate that endogenous SMOC2 blocks injury-induced jaw bone osteonecrosis and offsets age-induced periodontal decay.

## Introduction

Growing cells develop amidst a secreted fibrotic extracellular environment, providing not only structural support, but also having a bioactive role promoting growth and cell attachment. Here we investigate the functional roles of SMOC2 (SPARC related modular calcium binding 2 protein), a member of the secreted protein acidic and rich in cysteine (SPARCs) family of matricellular proteins. Matricellular proteins are non-structural components of extracellular matrix (ECM) often involved in regulating growth factor signaling^1^ and promoting wound repair^2^. Some matricellular proteins can regulate craniofacial development. One such example is periostin, which acts in the jaw to maintain periodontium structural integrity to ensure tooth anchorage^3^. Recent findings in several species suggest SMOC2 plays a more widespread role regulating craniofacial formation. *Smoc2* knockdown in zebrafish produces severe craniofacial hypoplasia^4^, canine SMOC2 reductions correlate with breed-specific brachycephaly^5^, and human *SMOC2*-mutated patients display both maxilla and mandible defects, and alveolar bone reductions potentially producing tooth defects^6^. In zebrafish, *smoc2* knockdown reduces expression of bone morphogenetic protein (bmp) target genes^7^. In vitro studies also suggest SMOC2 promotes keratinocyte attachment^8^, modulating focal adhesions and actin stress fibers by inducing integrins^9,10^. Its preferential enrichment in colon^11^, myeloid^7^, and dental^12^ stem cell lineages suggests functions in progenitor maintenance and/or cell anoikis^7,9,10^. Clinically, high SMOC2 levels correlate with poor cancer prognosis^13^ as they promote epithelial-to-mesenchymal transformation and induce clonal metastatic tumor cell growth with a strong angiogenic activity^9,10,14^. SMOC2 also induces TGF-β, in turn driving pathological fibrosis that often accompanies end-stage organ failure^15,16^. Strategies aiming at reducing SMOC2 levels have been suggested to have numerous therapeutic implications. Patients with homozygous *SMOC2* mutations have specific dental developmental defects. These include oligodontia (reduced tooth number), microdontia (small teeth), short roots, dentin dysplasia, and reduced alveolar/jaw bone density^12,17^. To investigate SMOC2 roles in a murine model, we generated a *Smoc2* inactivating mutation by introducing a targeted mutation in intron 1 splice donor site, thus mimicking a human deletion^12^. The resulting mouse mutants survive without early severe pathological consequences, somewhat similar to consequences of *SMOC2* mutation in children^12^. Our murine model predicts long-term pathological consequences. Like other matricellular proteins^2^, SMOC2 appears critical for the bone repair process. Following first molar avulsion injury, *Smoc2*^*−/−*^ mutants had impaired bone healing (mimicking osteonecrosis). Upon aging for a year, spontaneous age-induced periodontal bone and root loss are observed in *Smoc2*^*−/−*^ mutants. Strategies modulating the SMOC2-induced secretome might improve alveolar bone repair following a number of dental treatments. This could be applied clinically to alleviate jaw osteonecrosis-like symptoms^18^, a frequent decisive consequence of tooth extraction during bisphosphonate osteoporosis treatment.

## Results

### *SMOC2* mutation in a patient produces specific dental abnormalities and mild skeletal dysplasia

**Figure 1:**
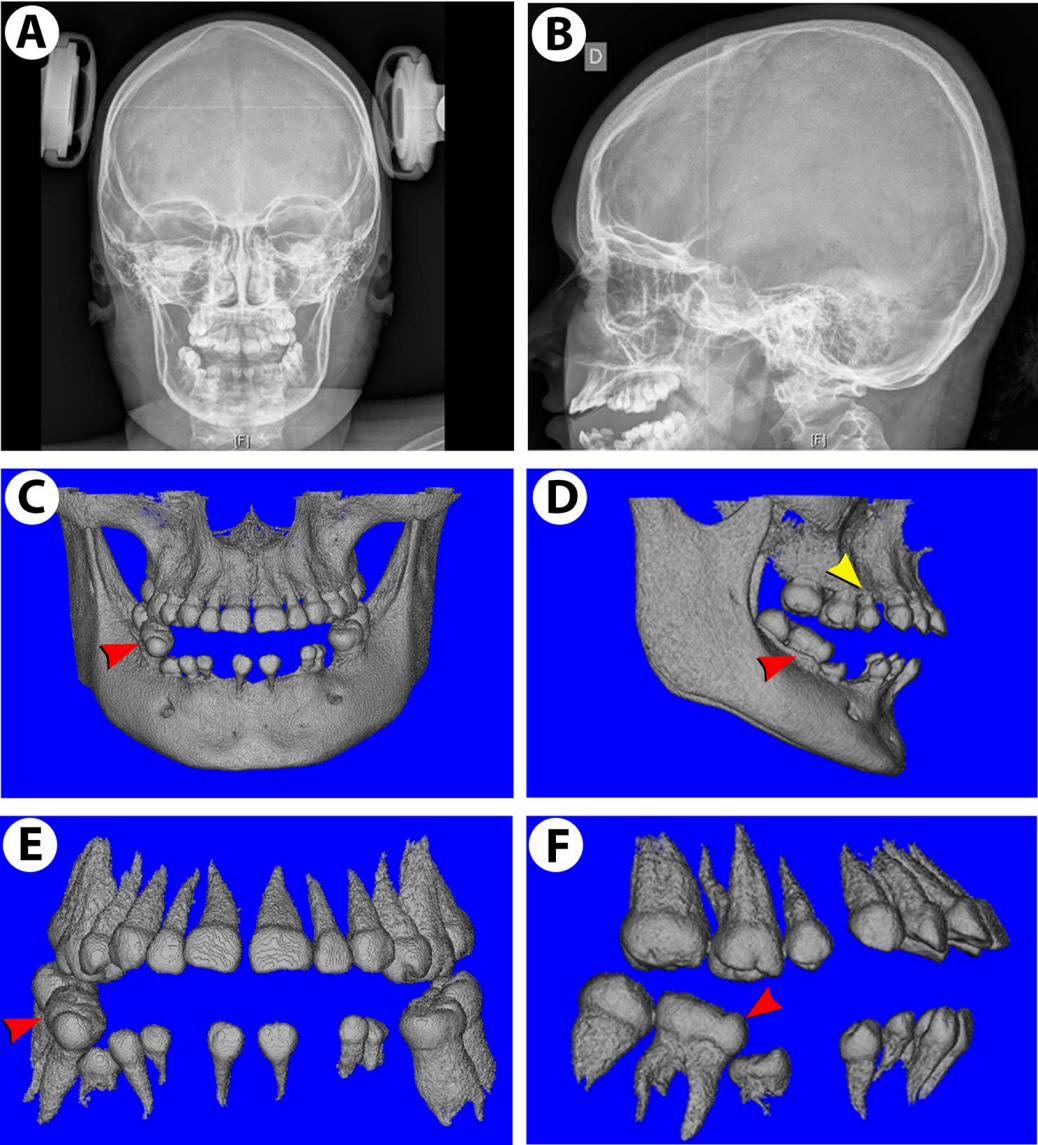
Radiographic and cone-beam computed tomography (CBCT) images of a 9 year-old girl with a homozygous *SMOC2* mutation (also see Ref.^12^). (**A**,**B**) Respective frontal and lateral radiographic views of the skull. Cranial bones and sutures, sella turcica, and orbit structures are normal except for the maxilla and mandible. (**C**) A full-frontal view of the dentition. The lower dentition displays more severe oligodontia and microdontia compared with the upper teeth. Affected teeth are typically 15–20% smaller than normal, except for the lower right first molar (red arrowhead), which is bigger. (**D**) Extracted CBCT images of full-lateral views. Defects include macrodontia (red arrowhead) and microdontia (yellow arrowhead). (**E**,**F**) Extracted frontal and lateral views with the lower dentition displaying more severe oligodontia and microdontia compared with the upper teeth. Image extraction of isolated teeth (using the Analyze 11.0 software) shows that a micro-root structure consistently accompanies microdontia. A false blue background was added using the Analyze 11.0 software (**C**–**F**), and the figure was labelled with Adobe Photoshop CS6.

We initially reported that SMOC2 deficiency severely disrupts tooth formation, as patients with a loss-of-function of this secreted matricellular protein display striking phenotypic defects in primary and permanent dentitions including microdontia, oligodontia, dysplastic root formation and alveolar bone hypoplasia^12^. Continual clinical monitoring of a 9 year-old affected girl showed similarly severe defects of permanent incisors, and a potentially progressing skeletal dysplasia^12^. While frontal and lateral radiographic views (Fig. 1A,B) of the skull showed no severe skull malformations, lumbar vertebrae were markedly flattened (displaying platyspondyly; Supplementary Fig. S1A–C,E) and the presence of a hyperlordotic curved spinal column suggested skeletal defects might worsen with age, just as wider iliac wings suggested a small degree of skeletal dysplasia (Supplementary Fig. S1D) not observed in other regions (Supplementary Fig. S1F). Tooth alveolar bone hypoplasia appeared generalized (Fig. 1A; Bloch-Zupan et al.^12^). For most teeth, cone-beam computed tomography (CBCT) imaging confirmed that upper (maxillary) teeth and corresponding alveolar bone were relatively less affected (Fig. 1C), compared with the mandibular dentition which presented severe oligodontia, microdontia and dysplastic roots (Fig. 1D). The right first permanent mandibular molar displayed however macrodontia (Fig. 1C,D, red arrowhead). Extracting CBCT images showed the relations of alveolar bone, tooth, and adjacent root structure. Full-frontal views showed severe dental anomalies in lower jaw with alveolar bone hypoplasia (Fig. 1E). Image extraction of isolated teeth showed that a micro-root structure consistently accompanies patient microdontia (Fig. 1E,F).

### *Smoc2*-*GFP* reporter analysis suggests fetal origins of SMOC2-deficiency defects

**Figure 2:**
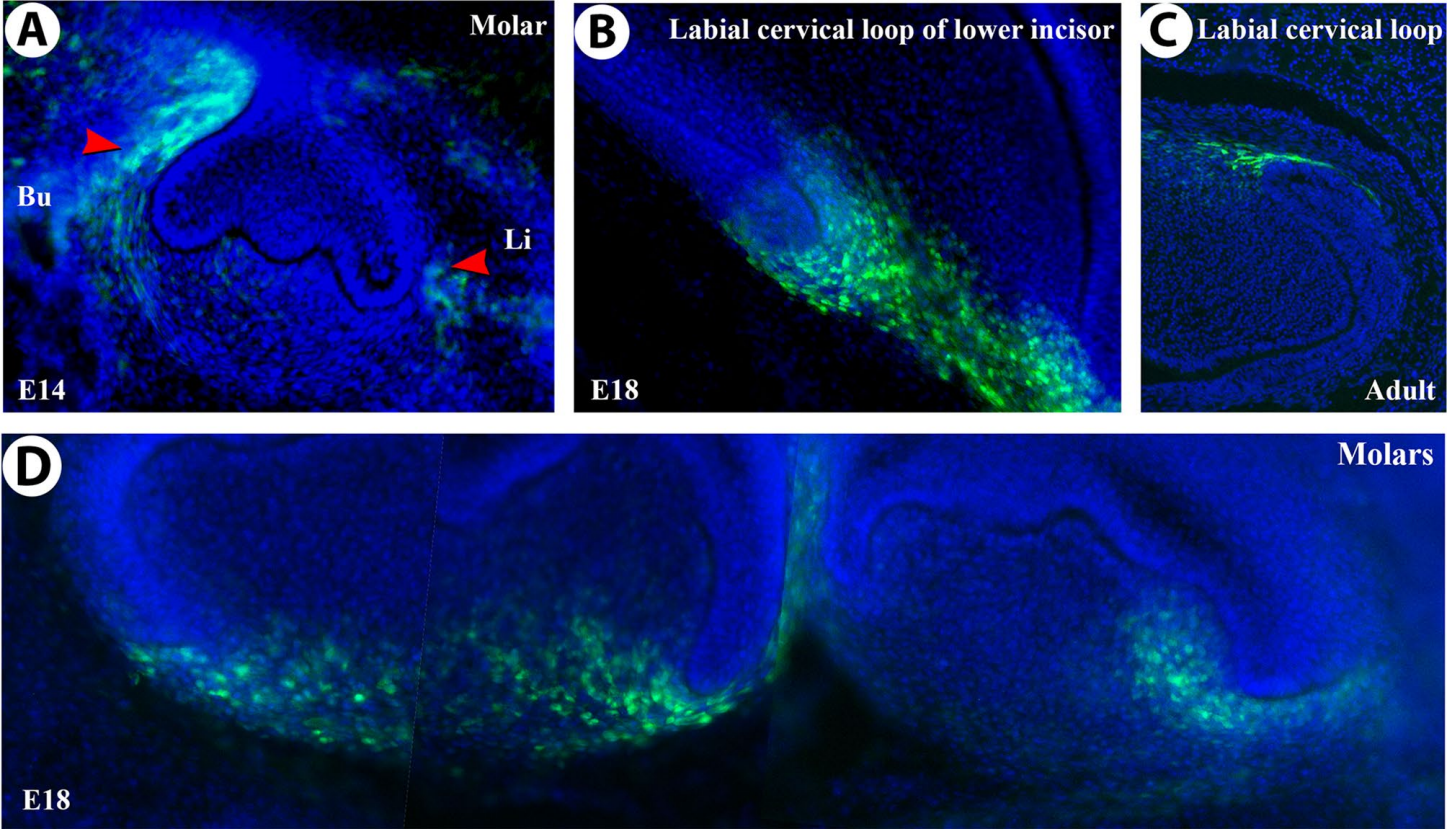
*Smoc2*-driven *GFP* immunolocalization in E14.5 (**A**) or E18.5 (**B**,**D**) developing teeth, and in adult mouse teeth (**C**). *Smoc2*-driven *GFP* is detected in the molar dental follicle mesenchymal populations surrounding the outer dental epithelium (**A**, red arrowheads), and the mesenchyme surrounding labial cervical loop of the lower incisor at fetal (**B**) and adult stages (**C**). *GFP* expression in E18.5 first and second molar mesenchyme (**D**) is also in the mesenchyme surrounding molars. Twelve fetal E14.5 and E18.5 and six adult (7 week-old) samples were used for each analysis. *Bu* buccal side, *Li* lingual side. This, and all following figures were labelled with Adobe Photoshop CS6.

To understand the physiopathology of these dental and mineralized tissues anomalies, we turned to mouse models. While *Smoc2* expression at late (fetal) stages of mouse development suggested its disruption could produce tooth growth deficiencies^12^, by performing lineage tracing using a heterozygous *Smoc2* modified allele with an in-frame *green fluorescent protein* (*GFP*) reporter insertion^11^ we confirmed the presence of this reporter in dental progenitor cells. In embryonic (E)14.5 *Smoc2*-*GFP* mice, GFP immunostaining labeled first molar dental follicle mesenchymal populations (Fig. 2A, red arrowheads). At E18.5 and post-natally, GFP was highly enriched in the mesenchyme surrounding the labial and lingual cervical loops of the lower incisor (Fig. 2B,C) and molar cervical loops (Fig. 2D), but absent from epithelial cervical loop stem cell zones allowing rodent incisors to continuously grow and produce enamel^19^. Other GFP-labeled sites at fetal stages were the telencephalon (Supplementary Fig. S2A), perioptic mesenchyme (Supplementary Fig. S2B, red arrowhead), basal nasal epithelial cells (Supplementary Fig. S2C), and hair follicle vibrissae (Supplementary Fig. S2D). Altogether, *GFP* analysis showed dynamic *Smoc2* expression often localizing to proliferative zones with a property of continuous self-renewal, like the intestinal crypts^11^.

### A novel mouse *Smoc2* mutation leads to reduced molar size and ectopic molars

To further investigate SMOC2 function, we generated a mouse mutant with a targeted deletion of *Smoc2* second exon, predicted to lead to a loss-of-function (Supplementary Fig. S3A; Supplementary Table S1). Mice harboring the conditionally mutated allele were generated, and were crossed with CMV-*Cre* transgenic mice^20^ to obtain in vivo excision of the floxed *Smoc2* exon. Mice homozygous for the exon 2-deleted allele (knock-out allele, Supplementary Fig. S3A) were found to be viable and obtained at a correct Mendelian rate. No gross embryonic (E9.5, E12.5) or fetal (E14.5–18.5) malformations were observed in *Smoc2*^*−/−*^ mutants. RT-PCR analysis comparing wild-type (*WT*) and *Smoc2*^*−/−*^ E14 incisor tooth buds indicated an absence of *Smoc2* mRNA in the knock-out mutants (Supplementary Fig. S3C), with no compensatory increases in *Smoc1* mRNA (Supplementary Fig. S3D) − *Smoc1* being a homologous gene whose inactivation produces lethality with severe ocular and limbs malformations^21^.

**Figure 3:**
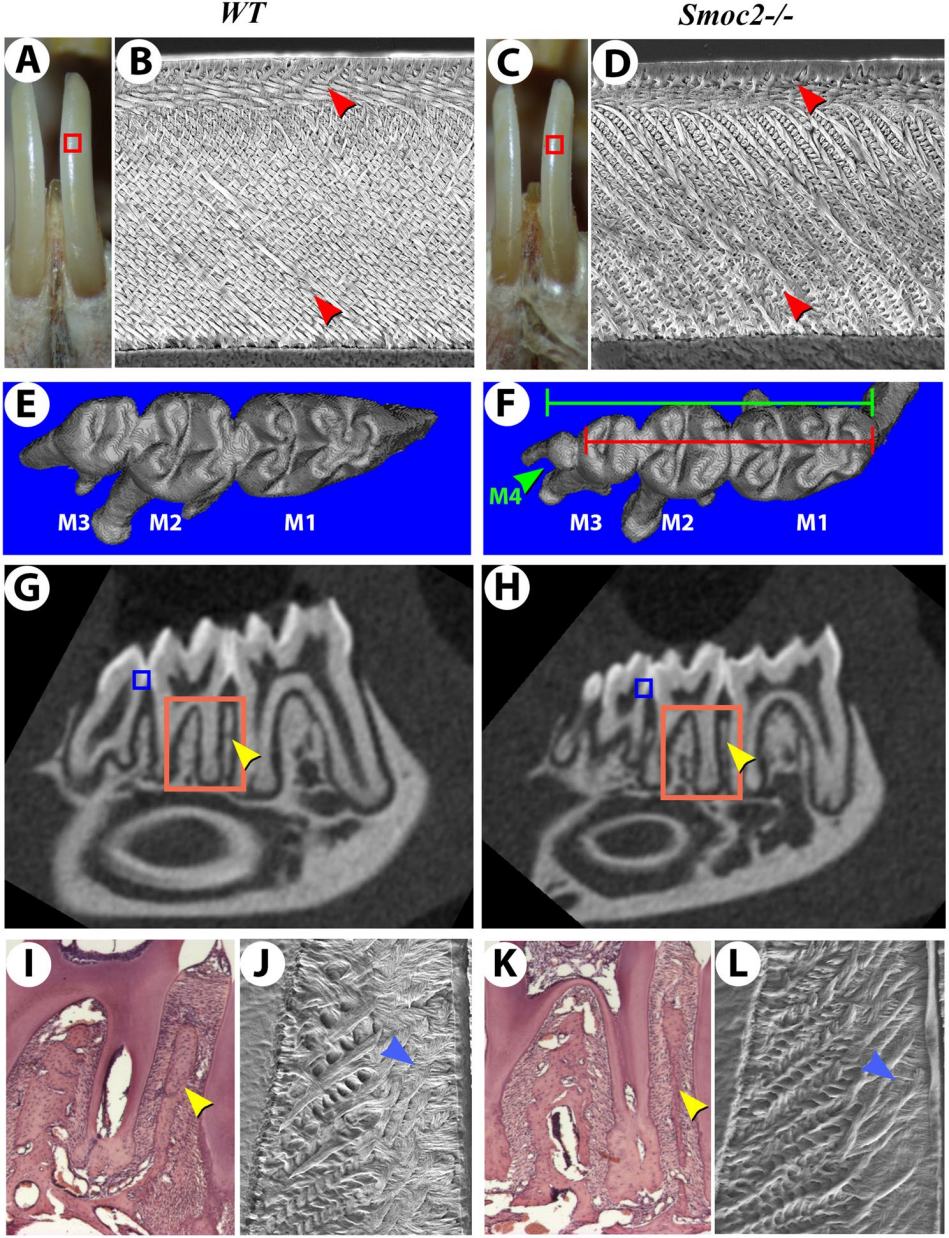
Dental alterations in *Smoc2*^*−/−*^ mutant mice. Seven week-old *WT* (**A**) and *Smoc2*^*−/−*^ mutant (**C**) lower incisors, the latter exhibiting slight size reductions. Scanning electron microscope (SEM) views of *WT* (**B**) and *Smoc2*^*−/−*^ (**D**) incisors. (taken from the red boxed regions in panels (**A**,**C**)) show mutants have alterations in both outer and inner enamel prisms structure (red arrowheads). (**E**,**F**) μCT 3D renderings of the occlusal surface of all three lower molars to assess morphological defects. Mutants display a significant reduction in the 1st–3rd molar field length (compare green and red bars in F: *WT* vs. mutant overall molar field length). Additionally, an ectopic distal 4th molar is observed in a fraction of *Smoc2* mutants (**F**, green arrowhead)*.* (**G**,**H**) μCT-derived mid-sagittal virtual sectioning indicates thinner alveolar bone in *Smoc2*^*−/−*^ mutant (**H**, yellow arrowhead) vs. *WT* (**G**, yellow arrowhead). (**I**,**K**) Standard histology (areas corresponding to those indicated by orange boxes in **G**,**H**) shows *Smoc2*^*−/−*^ mutants have reduced alveolar ossification (yellow arrowheads). (**J**,**L**) SEM analysis (region comparable to the blue-boxed region in **G**,**H**) shows reduced enamel crystal compaction, with irregular alignments in *Smoc2*^*−/−*^ mutant (blue arrowheads).

On a gross level, skeletal morphology appeared normal at fetal and perinatal stages (Supplementary Fig. S4), but detailed examination revealed specific defects. Enamel (the hardest mineralized tissue) of mouse teeth normally has an orange/yellow color, whereas in *Smoc2*^*−/−*^ mutants it had a whiter appearance (Fig. 3A *WT*; 3C *Smoc2*^*−/−*^), suggesting structural impairment. Scanning electron microscopy (SEM) confirmed irregular alignments and reduced compaction of enamel crystals in *Smoc2*^*−/−*^ mutants (Fig. 3B,J *WT*; 3D,L *Smoc2*^*−/−*^)*.* We performed X-ray micro-computed tomography (μCT) imaging and surface rendering to assess tooth defects. Both incisor and molar number was normal in most of the mutants analyzed, but discrete cusp morphology changes were observed, still allowing normal dental occlusion (Fig. 3E,F; Supplementary Fig. S5A,B,E,F). Two specific dental anomalies were observed, namely a reduction of the 1st–3rd molar field length (Fig. 3E,F: green bar [*WT*] vs. red bar [*Smoc2*^*−/−*^]: ~ 12% reduction, n = 83 mice analyzed, *p* value < 0.01), and a distal mandibular ectopic 4th molar found in 25% of *Smoc2*^*−/−*^ mutant females and 16% of mutant males (Fig. 3F, green arrowhead, Supplementary Table S3). While typically rodent ectopic 4th molars arise in the diastema^22,23^, *Smoc2*^*−/−*^ ectopic molars are exclusively distal (adjacent to 3rd molars), similar to *Wnt10a* mutants also displaying supernumerary mandibular 4th molars^24^. Roots are also consistency reduced in size, displaying taping shorter shapes (Supplementary Fig. S5C,D,G,H). Additional variations of phenotype are shown in Supplementary Fig. S6. μCT scan screening also indicated alveolar bone defect in 7-week-old *Smoc2*^*−/−*^ mutants (Fig. 3G *WT*; 3H *Smoc2*^*−/−*^), with reduced alveolar ossification confirmed by standard histology (Fig. 3I *WT*; 3 K *Smoc2*^*−/−*^).

### mRNA-seq analysis to assess *Smoc2* targets in bone and tooth

To assess mRNA changes resulting from *Smoc2* knock-out, high throughput mRNA-sequencing (mRNA-seq) analysis was performed on two types of sample preparations, in the aims of identifying any SMOC2 targets with critical roles in fetal tooth growth or bone regenerative response. In a first experiment, RNA was extracted from 6 litter-matched female E14.5 first molars from each group, then analyzed. Fetuses were genotyped for *Smoc2* or *Sry* (a male-specific transcript) to identify *Smoc2*^*−/−*^ and *WT* samples (Supplementary Table S1). Female *Smoc2 *^*−/−*^ mutants display a higher rate of 4th molar duplications (25% vs. 16% in males, see Supplementary Table S3), hence female samples were used to increase the potential to uncover tooth patterning targets. First molars were selected as these could be readily dissected at these early stages in tooth formation. RNA-seq data (Supplementary Table S4 lists relevant down-regulated transcripts) confirmed the down-regulation of *Smoc2* transcripts, and down-regulation of other gene transcripts including *bone gamma-carboxyglutamate protein* (*Bglap*), that could potentially affect bone turnover^25^, and *dentin matrix acidic phosphoprotein 1 (Dmp1)* potentially causing dental and osteogenic differentiation defects^26^ or dentin dysplasia^12^. Reduction of *odontogenic ameloblast-associated protein* (*Odam*), encoding a secreted protein guiding early enamel formation, could perturb enamel compaction in *Smoc2*^*−/−*^ mutants (see Fig. 3).

mRNA-seq analysis was also performed on E18.5 microdissected mandibular bone. This analysis (Supplementary Table S6) revealed a down-regulation of *dentin sialophosphoprotein* (*Dspp*), a major dentin extracellular matrix protein instructing dentin, root, and periodontal growth^27^ and important for craniofacial development. RNA changes were analyzed through the Gorilla (Gene Ontology enRIchment anaLysis and visuaLizAtion) tool to uncover potential developmental, growth, or tissue renewal-related alterations (Supplementary Figs. S7, S8). The most highly enriched gene ontology pathway at E18.5 (GO:0051707) was “response to other organisms” (Supplementary Fig. S8), including *Cxcl13*, *Defb14*, and *Gsdmc* as targets (Supplementary Tables S6, S7) altering cytokine signaling and bone healing^28^, targets potentially involved in both mouse and patient tooth and skeletal alterations and deficits in healing response. We are currently examining mechanisms to understand these targets.

### SMOC2 is required for bone healing to regulate inflammatory response

**Figure 4:**
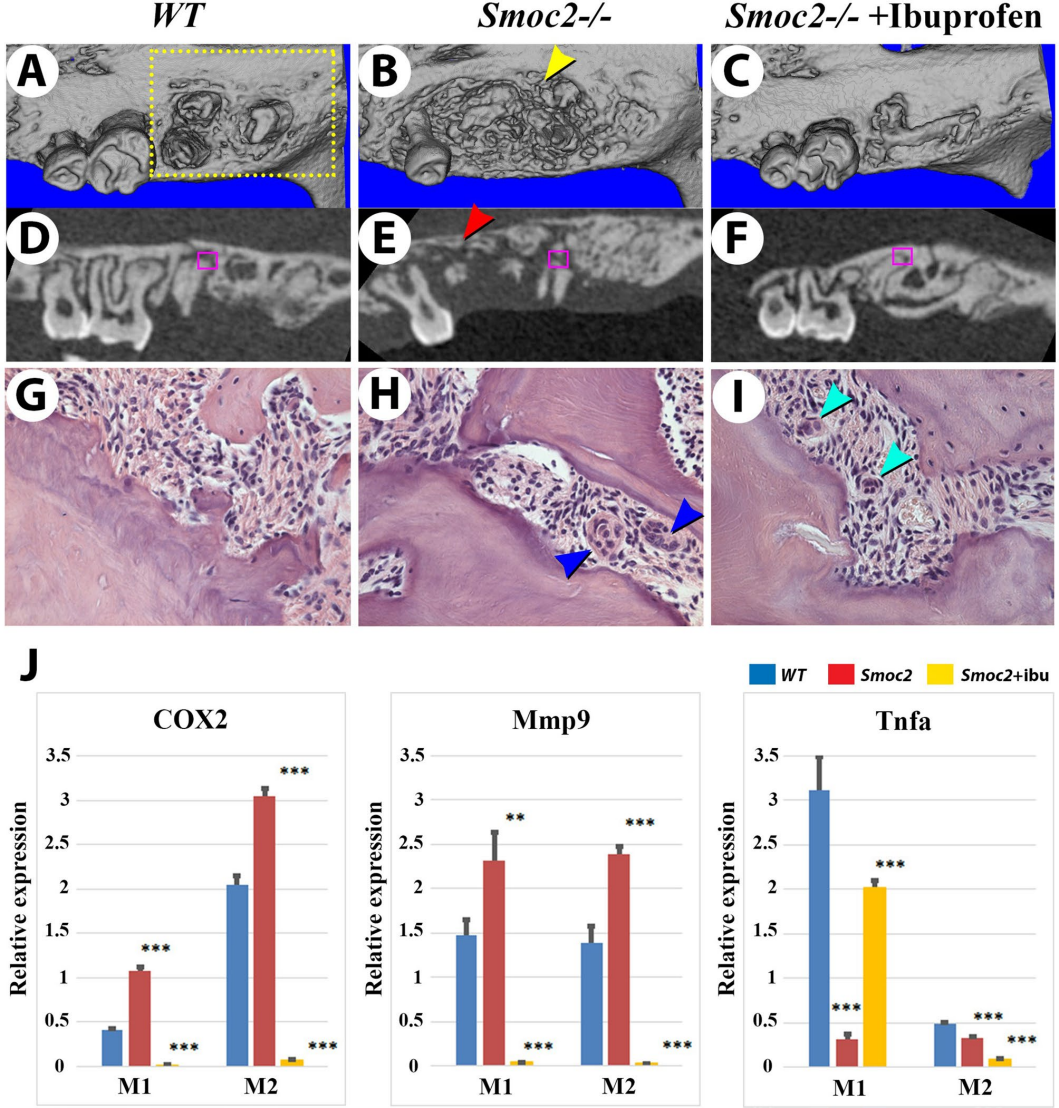
Increased bone loss and osteoclast activation in post-extraction *Smoc2* mutants. Six-week post-extraction μCT images of two months-old *WT* (A, D) and *Smoc2*^*−/−*^ (B, E) males show delayed healing and an extensive resorption around the extracted first molar (yellow arrowhead) and around 2nd molar (red arrowhead) in the *Smoc2*^*−/−*^ mutant. Bone loss defects in *Smoc2*^*−/−*^ mutant are rescued by short-term ibuprofen treatment (30 mg/kg for 10 days following surgery) (**C**,**F**). (**G**–**I**) Histological images near resorbed roots (corresponding to purple-boxed regions in **D**–**F**, respectively) show multinucleated cells (potentially osteoclasts), more pronounced in *Smoc2*^*−/−*^ mutant (**H**, blue arrowheads), and smaller in the *Smoc2*^*−/−*^ ibuprofen-treated specimen (**I**, green arrowheads). Three independent samples were analyzed for each group. (**J**) RT-PCR analysis of M1 or M2 molars and adjacent periodontal tissue shows relative expression levels of *Cox2*, *Mmp9*, and *Tnfa* normalized to *Gapdh*. *Cox2* and *Mmp9* mRNA are increased, while *Tnfa* is decreased 7 days post-extraction in *Smoc2*^*−/−*^ mutant in both M1 and M2, in comparison to *WT*. Both *Cox2* and *Mmp9* are drastically reduced in *Smoc2*^*−/−*^ mutants treated with ibuprofen. Each sample was obtained from 7 mice and done 3 times repeatedly. Statistical analysis was performed using Student’s t test: *p value < 0.05; **p value < 0.01; ***p value < 0.001.

Although long-term monitoring of *Smoc2*^*−/−*^ mutant mice (up to 18 months) showed comparable survival to *WT*, non-significant reductions in body weight were observed. This was similar to reports at the International Mouse Phenotyping Consortium for a similar C56BL/6 *Smoc2* loss-of-function^29^. Upon tail-clipping for genotyping, we observed more truncated tails (compared to *WT*), suggesting injury-induced growth deficits (data not shown). Tail growth requires a combination of cartilage, bone, vascular, and skin repair-not amenable to investigate which target tissue might produce defects. To specifically test the capacity of dental alveolar bone to heal, we used an experimental maxillary molar avulsion injury model. Two-month-old males were employed to avoid potential effect of estrogen variations on bone healing found in females. *Smoc2*^*−/−*^ mutants and age-matched controls were deeply anesthetized before the left 1st maxillary molar crown was removed (leaving the underlying molar roots intact) creating an experimental alveolar bone lesion. Six-week post-injury, μCT scanning images revealed that *Smoc2*^*−/−*^ mutants exhibited extended bone loss around the injured first molar compared to controls (Fig. 4A,D *WT*; 4B,E *Smoc2*^*−/−*^*,* yellow arrowhead). Bone loss in mutants included extensive 1st, and 2nd molar roots resorption. This appeared as a sort of accelerated “osteonecrosis” phenotype propagated as 2^nd^ molar root resorptions in 5 out of 7 mice (Fig. 4E, red arrowhead; for other representative images, see Supplementary Fig. S9). In 2 out of 7 mutants resorption of the 2nd molar crown was also observed. Alveolar bone/root mineral volume compared with total volume is tabulated in Supplementary Fig. S10, confirming significant reductions in jaw bone healing in *Smoc2*^*−/−*^ mutants.

As SMOC2 is activated by NF-κB signaling^9^, mutants could display alterations in inflammatory response and bone homeostasis. We tested if this differential bone loss (temporally correlating with the inflammatory stage of bone healing) could be prevented by a short (10-day) systemic treatment with a broad acting NSAID anti-inflammatory COX2 inhibitor, ibuprofen. Ibuprofen was provided for 10 days following surgery at a recommended pain-relieving dosage for mice (30 mg/kg body weight)^30^ to limit post-operative inflammatory response. Remarkably, augmented bone loss found 6 weeks after injury in *Smoc2*^*−/−*^ mutants disappeared after ibuprofen treatment. Hence, only 2 out of 7 mutants had a 2nd molar root resorption, with much less bone loss than untreated mutants (Fig. 4C,F, Supplementary Fig. S9). Histologically, at 6 weeks post-injury, groups of multinucleated osteoclast cells appeared at the resorbed roots in the remnants of mineralized tissue in *Smoc2*^*−/−*^ mutants (compare *WT* 4G vs. arrowheads in mutants in Fig. 4H,I: untreated and after ibuprofen treatment). Note that, in all *WT* animals analyzed (n = 7), ibuprofen treatment did not alter bone repair at 6 weeks as observed by μCT analysis (Supplementary Fig. S9).

### SMOC2 is induced after injury and its absence leads to increased osteoclast activity

To substantiate these results, we performed RT-PCR on RNA extracted at 7 days post-injury from the 1st and 2nd molar regions (tooth and periodontium), focusing on inflammatory-related signaling targets that might exhibit changes in expression in *Smoc2*^*−/−*^ mutants. We observed post-injury up-regulations of *cyclooxygenase 2* (*Cox2*) and *matrix metalloproteinase 9* (*Mmp9)* in *Smoc2*^*−/−*^ mutants, that were markedly reduced under ibuprofen treatment (Fig. 4J). Several other inflammatory signaling targets related to bone healing, including tumor necrosis factor α (*Tnfa*; Fig. 4J), *Integrin *α-D (*Itgad*), *CXC chemokine ligand 13* (*Cxcl13*), and *interleukin 17α* (*Il17a*) (Fig. S11) were decreased in both molars in *Smoc2*^*−/−*^ mutants after injury, with *Tnfa* expression being partially restored in M1 samples following ibuprofen treatments (Fig. 4J). An increased *RANKL (TNFSF11)*/*OPG (TNFRSF11B)* ratio indicates increased osteoclast activity^31^, as observed in *Smoc2*^*−/−*^ mutants 7 days following extraction (Supplementary Fig. S12). The *RANKL*/*OPG* ratio was largely reduced following ibuprofen treatment, suggesting treatments reduce osteoclast activation (Supplementary Fig. S12).

**Figure 5:**
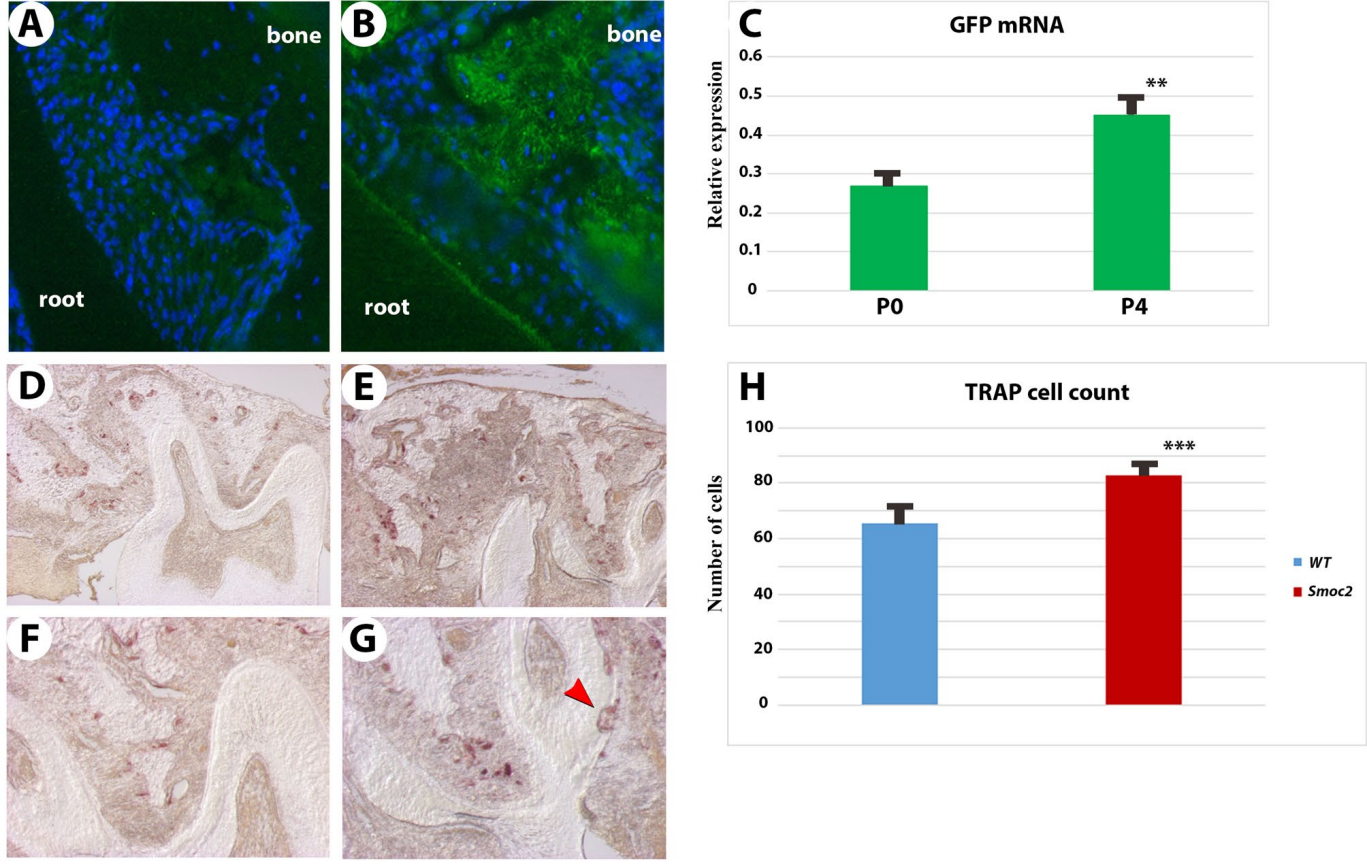
*Smoc2*-*GFP* reporter reveals *Smoc2* activation following injury. (**A**–**C**) In *Smoc2-GFP* reporter mice, *Smoc2*-driven *GFP* immunolocalization, weakly detected in the non-injured first molar, appears increased 4 days after molar extraction injury in adults. *Smoc2*-driven *GFP* is detected in periodontal tissue between root and bone (**A**), and is increased at 4 days after tooth injury (**B**). The increase in *GFP* was quantitated by RT-PCR, showing 40% higher *GFP* levels at day 4 (relative to *Gapdh*). (**D**–**H**) TRAP staining shows an increase of osteoclast cells 7 days after tooth injury in *Smoc2*^*−/−*^ mutants (**E**,**G**), especially around the M2 roots. The resorption of M2 root is observed in a *Smoc2*^*−/−*^ mutant with adjacent osteoclasts (**G**, red arrowhead). In the periodontal area, TRAP-positive cells were counted around the root-bone interface, five similar sections from each sample (magnification ×100) were captured and assessed using a HC2500 image analysis system (Fuji Photo Film, Tokyo, Japan) and the number of TRAP positive cells in each area was counted. Six independent samples were analyzed for each group.

Interestingly, both GFP immunofluorescence and RT-PCR confirmed increases in *Smoc2-Gfp* reporter expression at 4 days following tooth injury, suggesting that Smoc2 activation is part of a bone progenitor regenerative response (Fig. 5A–C). We also found that *Smoc2*^*−/−*^ mutants display increased osteoclast activity, as monitored by TRAP (tartrate resistant acid phosphatase) staining 7 days after injury around the surgical extraction site (Fig. 5D,F *WT*; 5E,G *Smoc2*^*−/−*^). TRAP staining indicated that osteoclasts were also increased at the 2nd molar roots site where the resorption of distal root was observed in *Smoc2*^*−/−*^ mutants (Fig. 5G, red arrow, quantitated in Fig. 5H).

### SMOC2 loss of function induces chronic periodontitis

**Figure 6:**
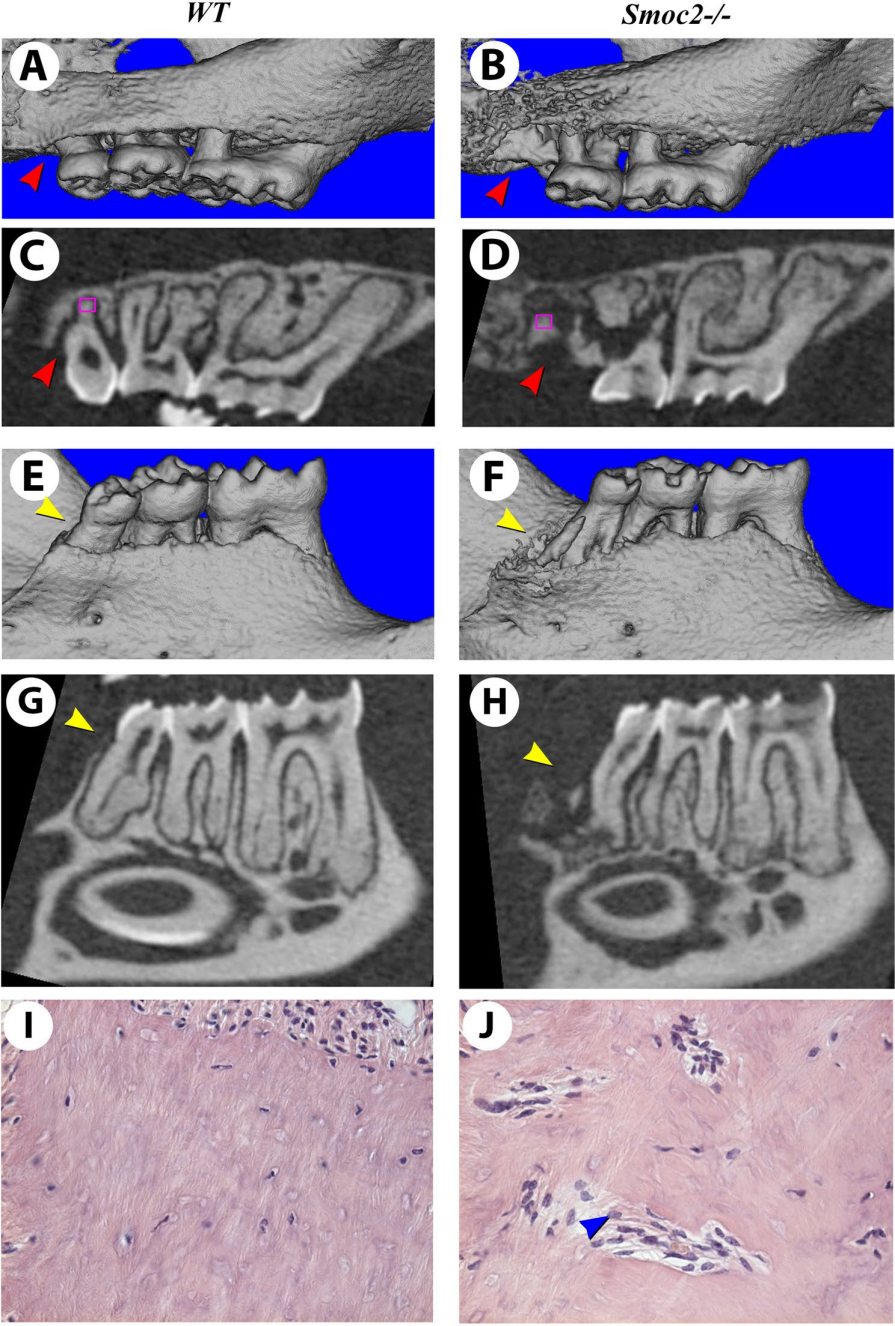
Periodontal bone loss in aging *Smoc2*^*−/−*^ mutants. One year-old *Smoc2*^*−/−*^ mutant mice have extensive spontaneous alveolar bone resorption. μCT 3D renderings of a 1 year-old *WT* (**A**) and an age-matched *Smoc2*^*−/−*^ mutant (**B**) show the resorption of alveolar bone and 2nd and 3rd maxillary molar roots in the *Smoc2*^*−/−*^ mutant (**A**–**D**, red arrowheads). The same resorptions are also found in mandibular 4th molars posteriorly to 3rd molars of *Smoc2*^*−/−*^ mutant (**E**–**H**, yellow arrowheads). Standard histology (of the regions indicated by purple boxes in **C**,**D**, respectively) shows that the *Smoc2*^*−/−*^ mutant has multinucleated cells infiltrated inside of the resorbed roots (**I**,**J**, blue arrowhead).

**Figure 7:**
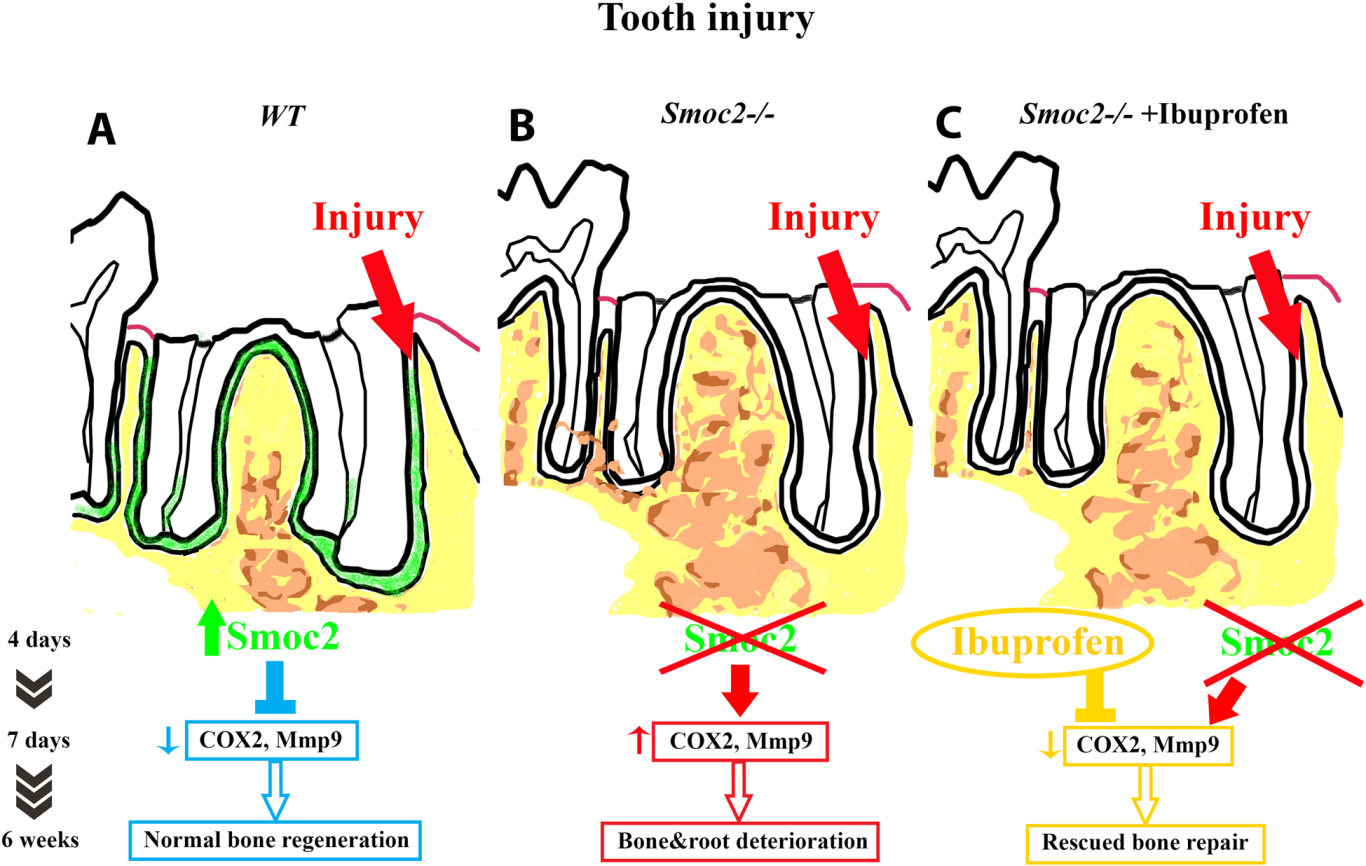
A model for the role of SMOC2 following tooth injury. In the normal (*WT*) situation (**A**), SMOC2 is induced 4 days after injury. The presence of SMOC2 reduces *Mmp9*, regulating over-activation of osteoclasts, and allowing reparative bone remodeling in a controlled effective manner. *Smoc2*^*−/−*^ mutants (**B**) display increased *Mmp9* and *COX2* expression, promoting osteoclast activation, leading to bone and root destruction during healing. When *Smoc2*^*−/−*^ mutants received a post-injury ibuprofen treatment (**C**), drug anti-inflammatory actions reduce *Mmp9* and *COX2*, a potential explanation for why *Smoc2*^*−/−*^ mutants display less bone and root deterioration after treatment. Figure from scanned hand-drawing, labelled using Adobe Photoshop CS6.

Augmented inflammation or defects in osteogenic or osteoclast-induced bone response could produce periodontal disease and alveolar bone loss—a predominant clinical consequence of chronic inflammation. To assess such possible defects, we performed μCT imaging on 1-year old mice, and found that ~ 40% of *Smoc2*^*−/−*^ mutants displayed spontaneous chronic periodontitis due to aging (n = 10, Supplementary Table S9). *Smoc2*^*−/−*^ mutants exhibited alterations characteristic of chronic inflammation marking periodontal disease. Thus, aging increased age-dependent bone and root resorption in the mutants (Fig. 6A,C,E,G *WT*; 6B,D,F,H *Smoc2*^*−/−*^*,* red arrowheads denoting loss of upper 3rd molar in mutants). Histological staining confirmed that *Smoc2*^*−/−*^ mutants have an increased number of multinucleated cells infiltrated inside of the resorbed roots (Fig. 6I *WT*; 6J *Smoc2*^*−/−*^, blue arrowhead), but no obvious differences in collagen fibers (Mallory's trichrome staining—Supplementary Fig. S13). We propose a coherent model explaining why *Smoc2* deficiency causes increased bone and root loss following tooth avulsion injury, and how ibuprofen might restore these changes (Fig. 7; see discussion).

## Discussion

### Mouse *Smoc2* mutants reveal accumulative effects of matricellular proteins in growth maintenance

We and others^11^ initially reported that the absence of SMOC2 in young mice did not dramatically alter skeletal patterning, nor disrupt organogenesis. One might interpret this as a limited (potentially redundant) role during normal development. Concurrent reports, though, indicated that SMOC2 promotes angiogenesis^14^, and increases TGF-β signaling regulating fibrosis^15,16^. Such effects might suggest SMOC2 roles in tissue maintenance, which could be offset during pathological fibrosis or cancer metastasis. Our data investigating aging and tooth injury response indicate a variety of stage- and cell type-specific SMOC2 functions. While SMOC2 overexpression in osteoblasts inhibits ossification^32^, we observe its deficiency produces age-dependent periodontal bone loss. Canine SMOC2 has been proposed as a key modulator of facial length, with reductions correlating with breed-specific brachycephaly^5^. In humans, *SMOC2*-mutated patients have maxilla and mandible defects that could be related to oligodontia and microdontia inducing alveolar bone reductions^6^. Mouse *Smoc2*^*−/−*^ mutants have a significant reduction in the size of the molar field, and also exhibit sporadic appearance of supernumerary molars, alterations in molar cusp patterning and smaller roots. We initially proposed facial changes could be secondary to reduced neural crest cell migration into the branchial arches^12^, as *Smoc2* knockdown in zebrafish produces severe craniofacial hypoplasia^4^. Deletion of the homologous *Smoc1* gene produces a severe overall growth deficiency, as well as limb syndactyly, and bone hypoplasia^21^. Other non-structural matricellular proteins, such as tenascins, are responsible when mutated for subtle tissue-specific phenotypes in neuroepithelial or osteogenic stem cell niches, and have been used to investigate pathological susceptibilities^33^.

### SMOC2 is required for bone repair and during periodontal aging

With aging, *Smoc2*^*−/−*^ mutant mice exhibit spontaneous alveolar bone deterioration and maxillary molar root resorption mimicking age-dependent dental and periodontal changes such as periodontal disease encountered in elderly human patients. We observed that short-term bone injury produces an osteonecrotic-like response in *Smoc2*^*−/−*^ mutants. Matricellular proteins like CCN1 aid wound repair by directly interacting with integrin receptors promoting tissue attachment^34^. SMOC2 induces integrin-induced stress fiber attachment^9^ and is required for a fibroblast to myofibroblast transition required for wound healing^15^. Injuries such as tooth extractions also induce an acute, highly regulated inflammatory response. Chronic inflammation produces increased bone loss in bisphosphonate-induced jaw osteonecrosis models^35^. *Smoc2* deficiency attenuates the inflammatory response and increase of interferon-γ (IFN-γ), tumor necrosis factor-α (TNF-α), and IL-1β levels caused by bleomycin injury-induced pulmonary fibrosis^16^ similar to reductions observed in alveolar bone after tooth avulsion injury (Figs. 4J; Supplementary Fig. S11). These reductions are detrimental to bone repair^36^. After wounding SMOC2 regulates early inflammatory response, until osteoclast activation carries out spatially limited bone degradation. SMOC2-dependent inflammatory cytokine and chemokine induction is followed by myofibroblast migration, collagen synthesis, and osteoclast-directed bone breakdown^16^. The early SMOC2-dependent pro-inflammatory response is necessary for post-extraction alveolar bone repair^37^. Both intramembranous and alveolar bone repair also require de novo coupling with angiogenesis^38^ wherein SMOC2 functions in vascular patterning may be augmented during injury-induced revascularization^14^. SMOC2 is differentially increased in failing human heart, potentially marking injury-induced myocardial ischemia and/or fibrosis^39^. *Smoc2* mutants could serve as a novel model to understand tissue repair, particularly in non-invasive therapy as anti-inflammatory (ibuprofen) treatment was shown to normalize defects in our experimental system.

### SMOC2 may normally block osteoclast “over-activation” during bone repair

Our results suggest that SMOC2 signaling is a key inflammatory regulator. SMOC2, like other SPARC matricellular proteins binds collagens which can affect matricellular ECM assembly and signaling. SPARC matricellular proteins have required functions during embryonic development. These roles persist in the adult, functioning as key clues for remodeling during inflammation and wound repair^40^. SMOC2 is rapidly reactivated during healing response, just as it is dysregulated during pathogenic fibrosis^15,16^. Evidence supports the premise that SMOC2 is a component of a pro-inflammatory secretome^10^. We show that aging *Smoc2*^*−/−*^ mutants exhibit periodontal bone loss—a sign of chronic inflammation. Injury triggers a highly abnormal cytokine/lymphokine response with *Smoc2*^*−/−*^ mutants displaying marked bone loss. A short term (10-day) systemic treatment with the NSAID ibuprofen restores bone regenerative response in mutants, this phenotypic rescue solidifying the connection with inflammatory signaling.

Collectively, our results suggest a fundamental role of SMOC2 in tooth/periodontium injury response illustrated in Fig. 7. In the normal (*WT*) situation, the presence of SMOC2 prevents over-activation of osteoclasts, lowering *Mmp9*, and allowing reparative bone remodeling in a controlled effective manner. *Smoc2*^*−/−*^ mutants display increased expression of *Mmp9*, an abundant oral endopeptidase showing increased expression during periodontal decay^41,42^. We find SMOC2 deficiency promotes osteoclast activation, increasing *Mmp9*, TRAP activity, and *COX2*, and leading to dysregulation of inflammatory cells and cytokines. We hypothesize that the acute inflammation response following tissue injury persists in *Smoc2*^*−/−*^ mutants, thereby damaging healthy surrounding tissues and causing further bone and root destruction^43^. Ibuprofen can diminish osteoclast number^44,45^, prevent excessive inflammation^46^, and reduce MMP9^47^. *Smoc2*^*−/−*^ mutants, given ibuprofen for 10 days after tooth injury, display normalized *Mmp9* and *COX2* expression, and “rescued” bone repair. Clinically, short-term ibuprofen treatment does not impair post-tooth extraction bone repair or is detrimental to dental implant osseointegration^48^. Recent studies even indicate NSAIDs can inhibit periodontal osteoclast activation following bacterial lipopolysaccharide-induced inflammation^49^. The effects of NSAIDs in bone repair need to be better defined^50^, in particular the potential positive effects of NSAID administration may be beneficial in chronic pathological situations such as long-term bacterial infection, chronic stress, and chronic force. SMOC2-regulated events required during alveolar bone repair and/or altered during periodontal bone decay could indeed be reactivated–and therapeutically controlled—in other progenitor cell populations, serving as potential clinical targets for further directed therapeutics in the aging population.

## Methods

### Patients

The patients with a homozygous *SMOC2* mutation were initially described^12^ as part of a French Ministry of Health National Program for Clinical Research, PHRC 2008 HUS (Strasbourg University Hospital) No. 4266 and in the INTERREG IV Offensive Sciences A27 “Orodental manifestation of rare diseases” EU funded (ERDF) project. The two cousins and their family members gave informed written consent and documents for the D[4]/phenodent registry, a Diagnosing Dental Defects Database [see www.phenodent.org, to access assessment form], which is approved by CNIL (French National commission for informatics and liberty, number 908416). The clinical study is registered at https://clinicaltrials.gov: NCT01746121 and NCT02397824, and with the MESR (French Ministry of Higher Education and Research) Bioethics Commission as a biological collection “Orodental Manifestations of Rare Diseases” DC-2012-1677 within DC-2012-1002 and was acknowledged by the CPP (person protection committee) Est IV December 11th 2012. They were examined and followed up at the Strasbourg University Hospital Reference Center for rare oral and dental diseases and imaging such as X-ray and computed tomography (CBCT) necessary to patient treatment were available.

### Immunofluorescence

A previously described *Smoc2-EGFP-ires-CreERT2* knock-in (*Smoc2-ki*) mouse line, in which a GFP cassette was introduced in frame with the translational start site of *Smoc2*^11^, was used to perform *GFP* immunofluorescence reporter analysis. E14.5 and E18.5 fetuses fixed in 4% paraformaldehyde (PFA) for 2 h, were embedded in melted Ultra-pure Low Melting Point agarose gel (Invitrogen, REF16520-100) at a concentration of 4% for E14.5, or 6% for E18.5 in Peel-A-Way embedding molds (Polysciences) prior to cutting using a Leica VT1000 vibratome (thickness 30 µm). Adult *Smoc2-ki* mouse heads were fixed in 4% PFA overnight, decalcified in 10% EDTA (room temperature for ~ 2 weeks), embedded in OCT (Shandon Cryomatrix embedding resin, Thermo Fisher Scientific), and cryosectioned (Leica CM3050 S) at 8 µm. After incubated in blocking solution (10% normal donkey serum in PBS-Tween20) for 30 min, the primary antibody (Anti-GFP antibody-ChIP Grade, ab290, Abcam) was added overnight. Secondary antibody (Donkey anti-Rabbit IgG Secondary Antibody, Alexa Fluor 488) and DAPI (Sigma-D9542, Sigma-Aldrich) staining were performed. Images were acquired with a Leitz DMRB fluorescence microscope.

### Quantitative real-time PCR (RT-PCR)

To quantitate gene expression changes in tooth and bone, RT-PCR was performed on age-matched RNA samples for either control or *Smoc2*^*−/−*^ mice. For this, total RNA was extracted with the RNeasy Micro-kit and amplified (1 μg per reaction) by real-time RT-PCR using SYBR Green Reagents (Qiagen). cDNA templates were generated using the Oligo-dT primed Superscript II kit (Invitrogen). SYBR Green incorporation into amplified PCR products was detected using a Roche 480 LightCycler. Primer sequences (listed in Supplementary Table S1) were obtained from “Harvard primer” website (https://pga.mgh.harvard.edu/primerbank/, Primerbank), or designed using the Primer3web program. *GFP* primers were previously reported^52^. Expression levels were normalized to glyceraldehyde-3-phosphate dehydrogenase (*Gapdh*) levels. Seven mice of each genotype were used for each gene tested for GFP and inflammation markers. Tests were performed in triplicate to confirm changes. Statistical analysis was performed by Student's t test.

### X-ray micro-computed tomography (μCT)

*Smoc2*^*−/−*^ mutant samples were fixed in 4% PFA for 10–14 days, washed in demineralized water, and screened for tooth and bone mineral density alterations by X-ray micro-computed tomography (μCT) imaging using the Quantum FX μCT Imaging System (Caliper Life Sciences). The threshold to scan bone and tooth was set up at 90 kV and 160 µA with “Fine” scan option using a pixel size of 10–80 μm. DICOM images were imported into Analyze software (v11.0; Biomedical Imaging Resource, Mayo Clinic, Rochester, MN, USA) for image reconstruction and bone mineral analysis.

### Scanning electron microscopy

The teeth of 8-week-old *Smoc2*^*−/−*^ mutants and respective controls dissected from alveolar bone were serially dehydrated in 100% ethanol, transferred into propylene oxide/epon resin (Epon 812, Euromedex, Souffelweyersheim, France) embedding, followed by sagittal sectioning and diamond paste polishing (Escil, Chassieu, France). Briefly, samples were etched with 20% citric acid for 2 min, rinsed, dehydrated into pure ethanol and dried. A HUMMER JR sputtering device was used to coat teeth with gold–palladium (Technics, CA, USA). Scanning electron microscopy was then performed using a Quanta 250 ESEM system (FEI Company, Eindhoven, The Netherlands) at an accelerating electron voltage of 5 kV.

### mRNA sequencing (mRNA-seq)

Total RNA from six female E14.5 molars, along with female E18.5 mandibular bone adjacent to the first molar was obtained from *Smoc2*^*−/−*^ mutants and their respective wild-type (*WT*) controls. Following RNA extraction with the RNeasy Micro-kit, mRNA-seq library preparation was achieved according to Illumina protocols. Sequencing for each group was performed in triplicate. Sequence mapping relative to the mm10/NCBI37 mouse reference genome were performed using Tophat. Only when unique aligned sequence reads were obtained was gene expression quantification performed using HTSeq-0.6.1 (described at https://www-huber.embl.de/users/anders/HTSeq/doc/overview.html). For each transcript, reads per exon kilobase of model per million sequence reads (RPKM) were then converted to raw read counts, then added for each gene locus. Data normalization was performed as described^53^ and resolved with the DESeq Bioconductor package. A Benjamini and Hochberg-based multiple testing model provided adjusted *p* values^54^. Transcripts alterations with a RPKM > 1, and an adjusted *p* < 0.05 were considered.

### Molar avulsion injury

On the day of wound creation (D0), seven 2–3 month-old male *Smoc2*^*−/−*^ mutants and age-matched control mice were deeply anesthetized with intraperitoneal administration of 9% ketamine HCl and 1% xylazine (10 μl/g body weight). A number 11 blade scalpel was used to loosen the maxillary left first molar. The tooth was then broken by removing the crown (leaving the roots intact) by rotating clinical forceps causing the top of the tooth to crack. After removal, the extracted crown was verified for integrity. All *Smoc2*^*−/−*^ mutants and age-matched controls exhibited no surgical complications following tooth extraction. At 6 weeks after extraction, seven mice per group were sacrificed and μCT performed. For NSAID treatment, seven *Smoc2*^*−/−*^ mutants and age-matched controls were given ibuprofen (Children’s Motrin) at 30 mg/kg in the drinking water for 10 days^30^. Molecular responses in bone and root underlying either the 1st or 2nd molar were determined by RT-PCR 7 days after crown extraction (~ 30 mice per group).

### Histological analysis and TRAP staining

Heads of 2-month-old *Smoc2* mutants and age-matched controls were fixed in 4% PFA overnight, rinsed, and demineralized in 10% EDTA^55^. After several water washes, serial dehydration was performed in a series of graded ethanol solutions, followed by Histosol^R^ clearing, and paraffin embedding at 60 °C. Staining of 8 µm sections with hematoxylin/eosin and Mallory’s stain was performed according to standard procedures. To characterize osteoclast activity, a tartrate resistant acid phosphatase (TRAP) labeling kit (387A, Sigma-Aldrich) was used on deparaffinized sections assayed 7 days following molar crown extraction following manufacturer’s instructions. In all experiments 6 adult mice for each group were used.

### Ethics statement

All methods and experimental procedures were entirely carried out in accordance with all relevant French guidelines and/or regulations and thus were reviewed and approved by the IGBMC institutional safety committee. For procedures involving mice, all animals were maintained and manipulated according to protocols in agreement with the French Ministry of Agriculture guidelines for use of laboratory animals (IGBMC protocol 2012-097) and with NIH guidelines, described in the Guide for the Care and Use of Laboratory Animals. The *Smoc2*^*−/−*^ mouse line was created at the Mouse Clinical Institute (ICS) using Cre and Flp methods^51^, as described in Supplementary Fig. S1 and Supplementary Table S1.

## Supplementary information

Supplementary information
