# Supplementary material for: Deficiency of the SMOC2 matricellular protein impairs bone healing and produces age-dependent bone loss
Source: Sci Rep. 2020 Sep 9;10:14817. doi: 10.1038/s41598-020-71749-6 (PMC7481257; doi:10.1038/s41598-020-71749-6)
Supplement: Supplementary file 1 — Supplementary information [file 41598_2020_71749_MOESM1_ESM.pdf]

Supplementary Information

Deficiency of the SMOC2 matricellular protein impairs bone healing and produces age-dependent bone loss

Supawich Morkmued[1-5], François Clauss[6-8], Brigitte Schuhbaur[1-4], Valérie Fraulob[1-4], Eric Mathieu[9], Joseph Hemmerlé[9], Hans Clevers[10], Bon-Kyoung Koo[10], Pascal Dollé[1-4,11], Agnès Bloch-Zupan[1-4,6,7,12*], and Karen Niederreither[1-4,6*]

[1]Developmental Biology and Stem Cells Department, Institute of Genetics and of Molecular and Cellular Biology (IGBMC), 1 rue Laurent Fries, BP 10142, 67404 Illkirch, France
[2]Centre National de la Recherche Scientifique, UMR7104, Illkirch, France
[3]Institut National de la Santé et de la Recherche Médicale, INSERM U1258, Illkirch, France
[4]Université de Strasbourg, Illkirch, France
[5]Faculty of Dentistry, Pediatrics Division, Preventive Department, Khon Kaen University, Khon Kaen, Thailand
[6]Université de Strasbourg, Faculté de Chirurgie Dentaire, 8 rue Ste Elisabeth, 67000 Strasbourg, France
[7]Hôpitaux Universitaires de Strasbourg, Pôle de Médecine et Chirurgie Bucco-Dentaires, Centre de Référence des Maladies Rares Orales et Dentaires, CRMR O Rares, Filière TETECOU, ERN CRANIO, 1 place de l'Hôpital, 67000 Strasbourg, France
[8] Regenerative NanoMedicine, INSERM UMR1260, FMTS, 11 rue Humann 67000 Strasbourg, France Hôpitaux Universitaires de Strasbourg
[9] Université de Strasbourg, INSERM UMR1121, Biomaterials and Bioengineering, 11 rue Humann, 67000 Strasbourg, France
[10]Hubrecht Institute, University Medical Center Utrecht, and University Utrecht, The Netherlands
[11]Université de Strasbourg, Faculté de Médecine, FMTS, 4 Rue Kirschleger, 67000 Strasbourg, France
[12]Eastman Dental Institute, University College London, London, UK

Figure S1

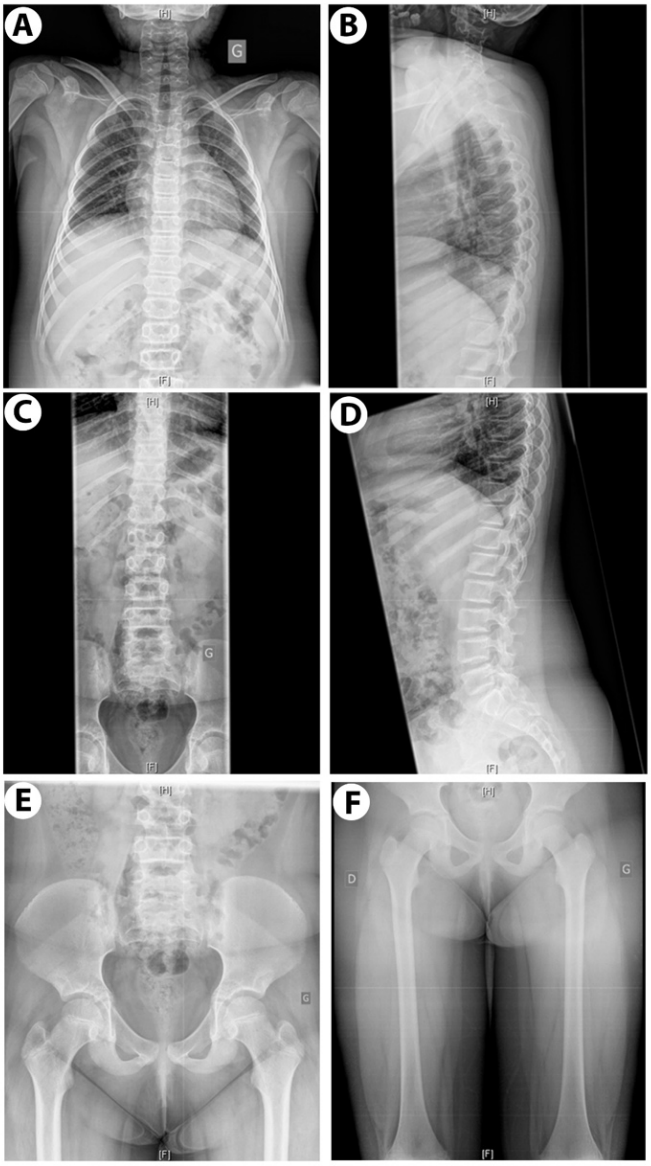

X-ray scans of *SMOC2* mutated patient. (A,B) shows no morphological abnormalities or structural alterations of cervical and dorsal vertebral bodies. (C,D) at the lumbar level, the patient displays a degree of platyspondyly, especially at lower lumbar level with a discrete posterior bi-concave deformation of the vertebral bodies. Lumbar hyperlordosis is also found. (E,F) shows broad aspects of the iliac wings, with relatively short and stocky femoral necks of normal volume.

Figure S2

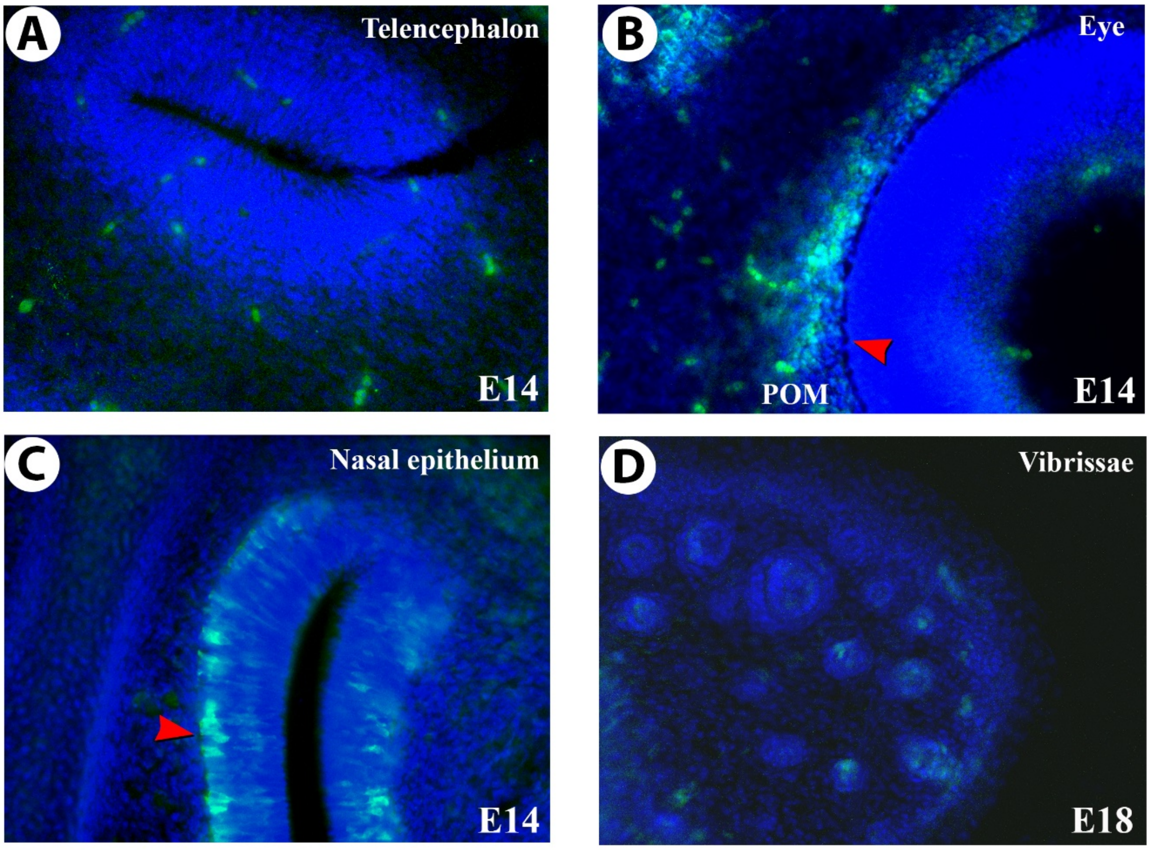

Smoc2-driven *GFP* immunolocalization on sections from E14.5 and E18.5 *Smoc2-ki* fetuses. *Smoc2*-driven *GFP* is diversely expressed in the E14.5 telencephalon (A), localized to E14.5 perioptic mesenchyme (POM) surrounding the developing eye (B, red arrowhead), and found in the basal cells of the nasal epithelium (C, red arrowhead). At E18.5, *Smoc2*-driven *GFP* also localizes to the vibrissae follicles (D). Images were acquired with a Leitz DMRB fluorescence microscope.

Figure S3

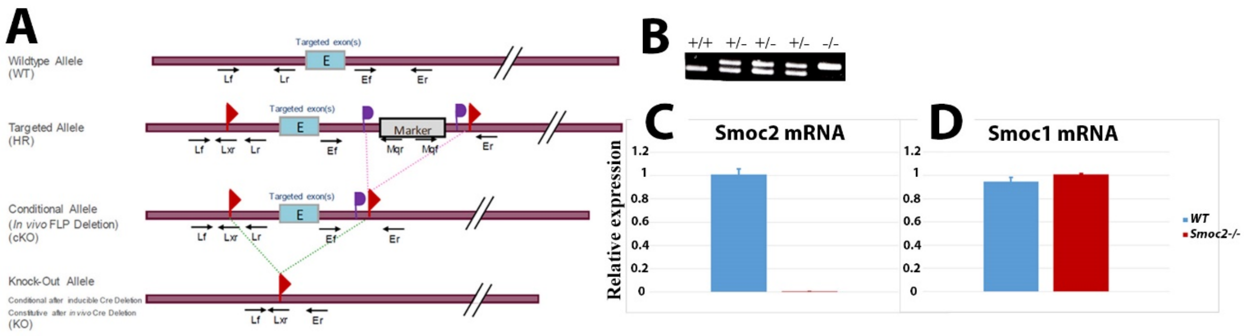

(A) Constructs and strategy used to generate a targeted mutation of the mouse *Smoc2* gene. The *Smoc2* (Ensembl gene ID ENSMUSG00000023886) locus was modified as shown to create the knock-out allele used in this study, with a targeted deletion of *Smoc2* second exon. After insertion of the targeting construct by homologous recombination (HR) in embryonic stem cells, mice were generated and, by successive crosses with *FLP*- and *Cre*-expressing transgenic lines, produced lines with a conditional allele (devoid of the selection marker, with a floxed targeted exon), and knock-out allele. Scheme adapted courtesy of the mouse clinical institute (ics-mci.fr). (B) shows an example of PCR genotyping of littermates from heterozygous breedings. Primer sequences are listed in the supplemental table. (C) shows RT-PCR analysis of embryonic tooth buds revealing non-detectable *Smoc2* mRNA in *Smoc2-/-* mutants, and (D) lack of a compensatory *Smoc1* upregulation. Primer sequences are provided in Table S1.

Figure S4

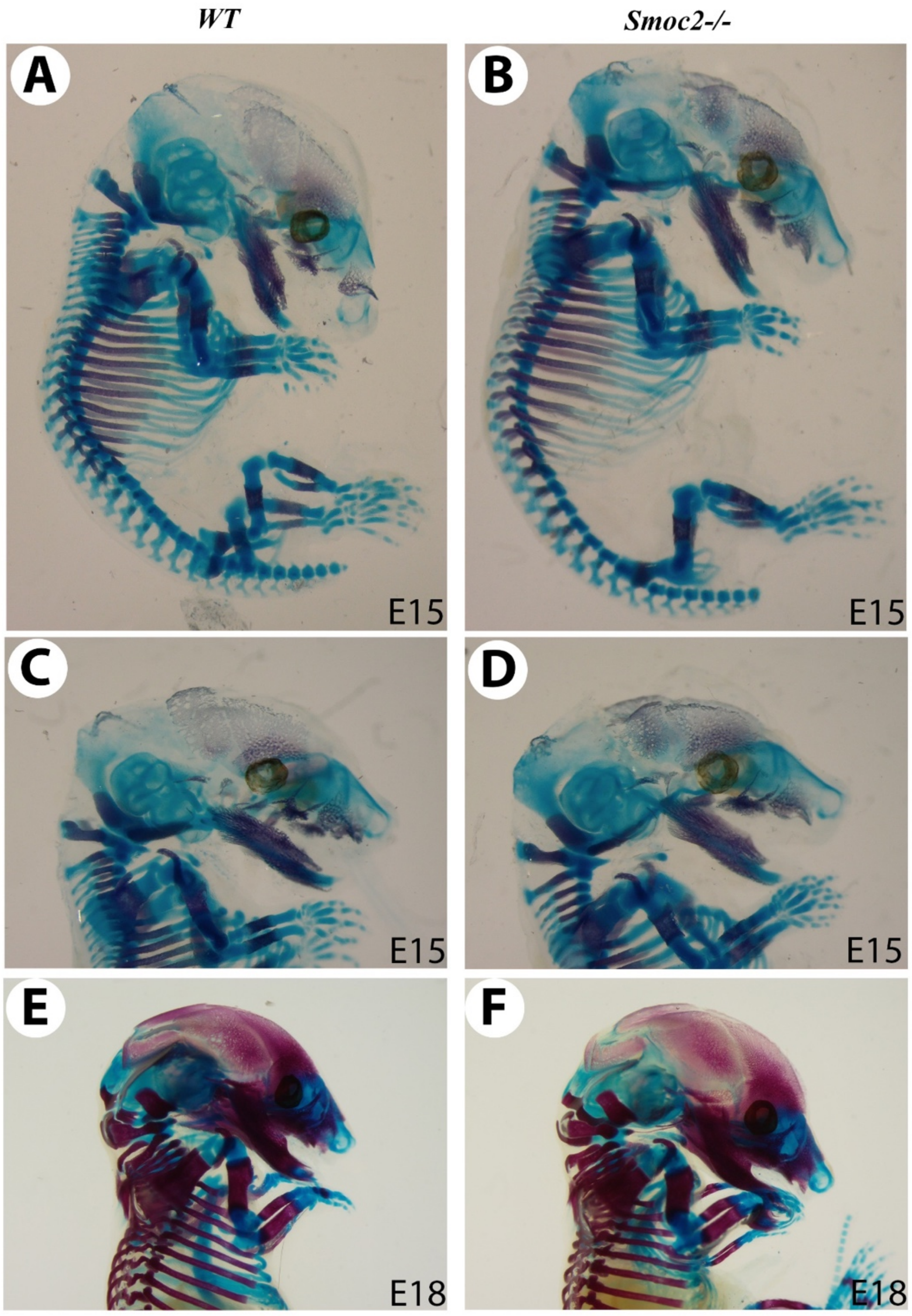

Skeletal analysis (alizarin red: bone, and alcian blue: cartilage staining) of E15.5 and E18.5 *WT* (A,C,E) and *Smoc2-/-* mutants (B,D,F). No obvious skeletal changes are seen in the mutants. Images were acquired with a Leica Z16 apochromatic microscope.

Figure S5

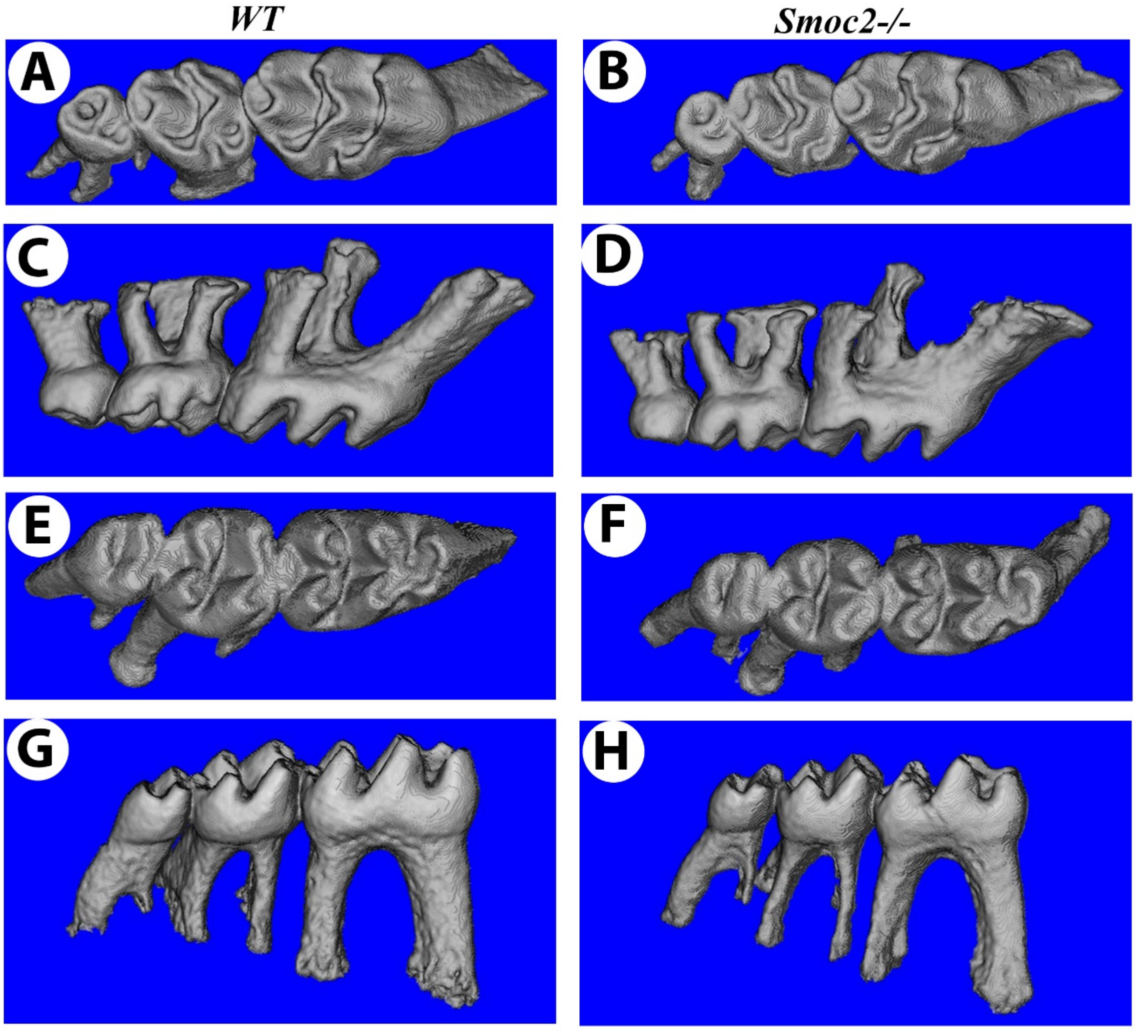

Molar cusp and root morphology (μCT imaging and surface rendering). *Smoc2-/-* mutants show slightly rounded upper (A,B) and lower molar cusp morphology (E,F), along with molar size reduction (C,D, G,H). Roots are also consistency reduced in size, displaying taping shorter shapes. The root anatomy changes shown in (H) would lead to a thinner septal bone and wider furcation bone in *Smoc2-/-* mutants. 3-D renderings are obtained from μCT data using the Analyze 11.0 software, including the addition of a false blue background to A-H.

Figure S6

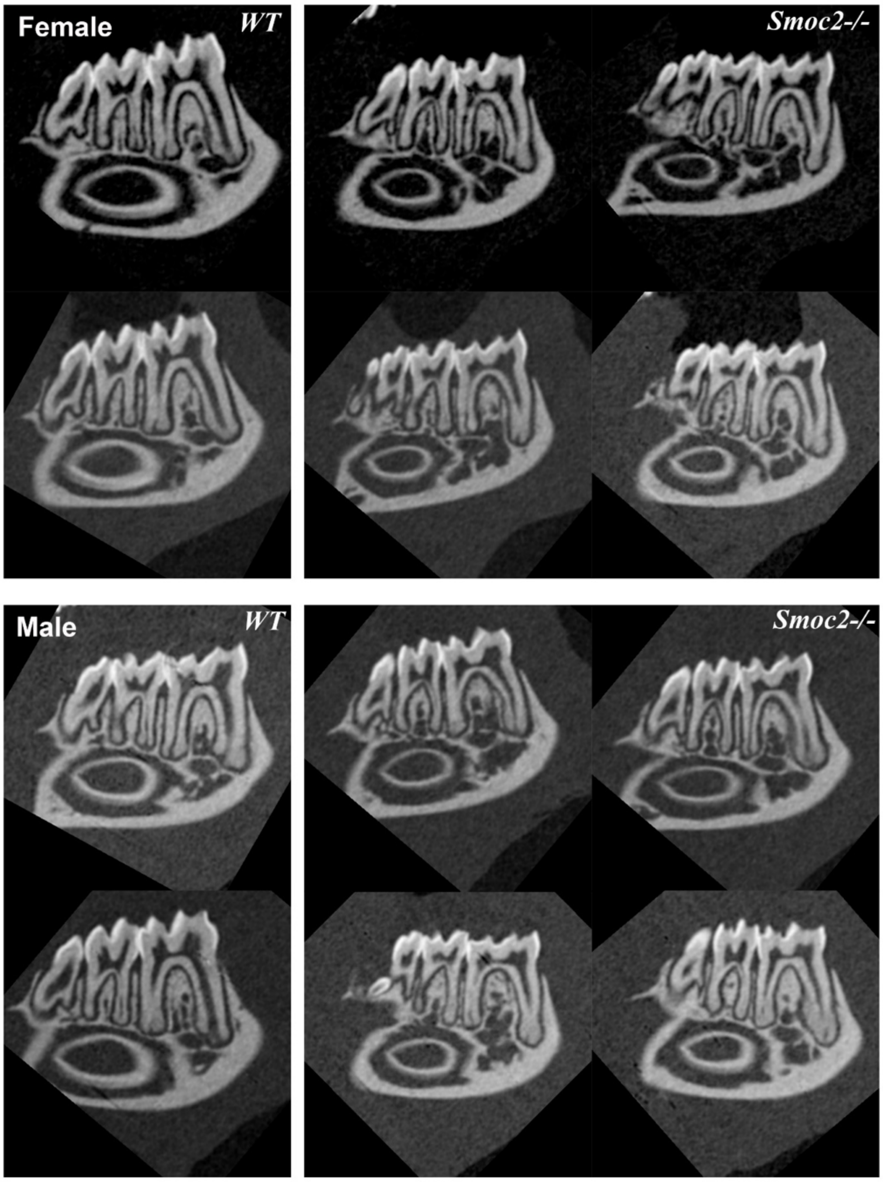

Variations in *Smoc2-/-* mutant tooth phenotypes (µCT virtual sections). Both female and male *Smoc2-/-* mutants show lower molar and lower incisor size reductions. A supernumerary 4th molar is at times observed in *Smoc2-/-* mutants with a slightly higher prevalence in females (25% in female and 16% in male mice). Images are obtained from µCT virtual sections.

Figure S7

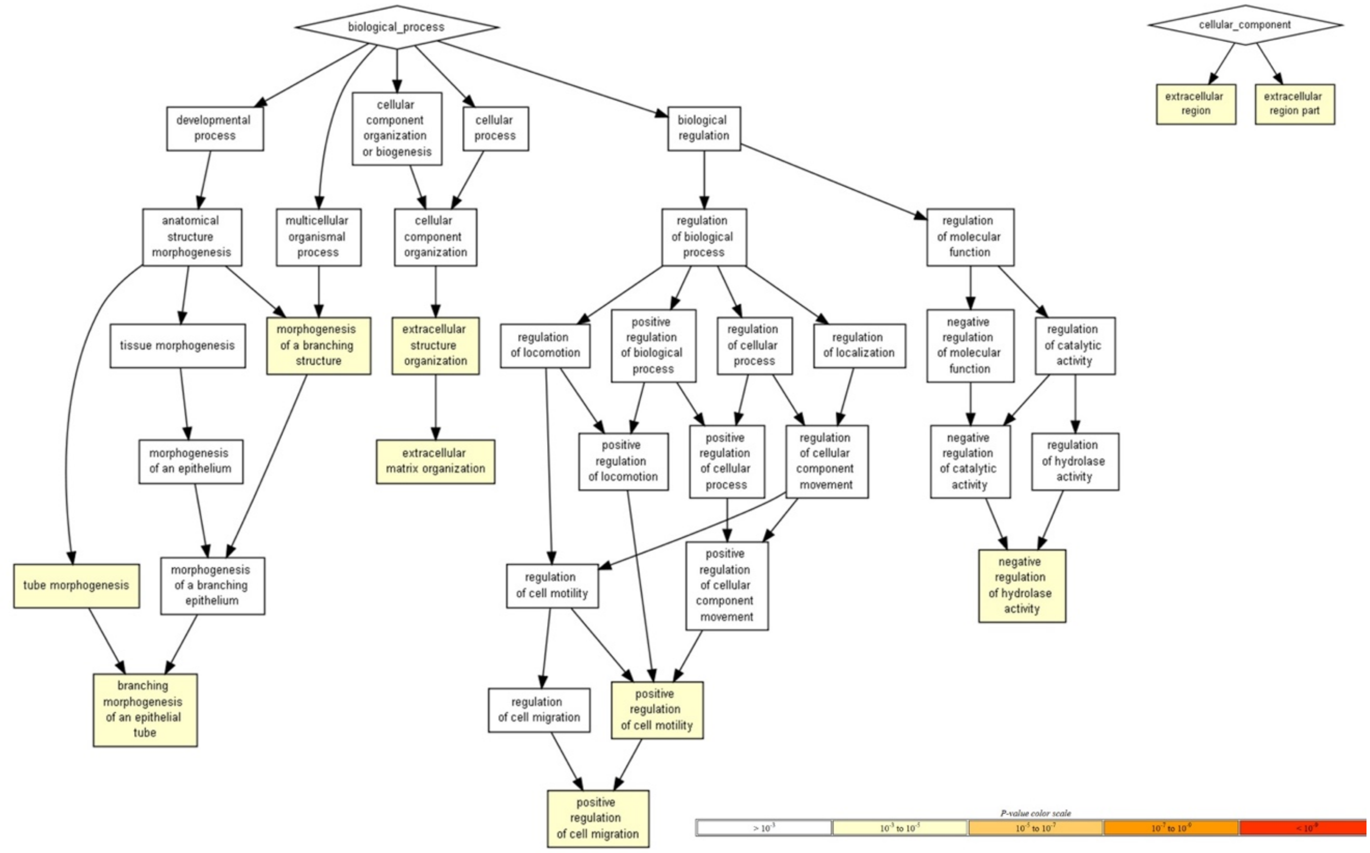

Gene Ontology enRIchment anaLysis and visuaLizAtion tool (Gorilla) was used to analyze RNA-seq data from E14 molars. The resulting enriched gene ontology (GO) terms are ranked in lists of significantly changed pathways (yellow-colored boxes indicate a *p*-value $<10^{-3}$).

Figure S8

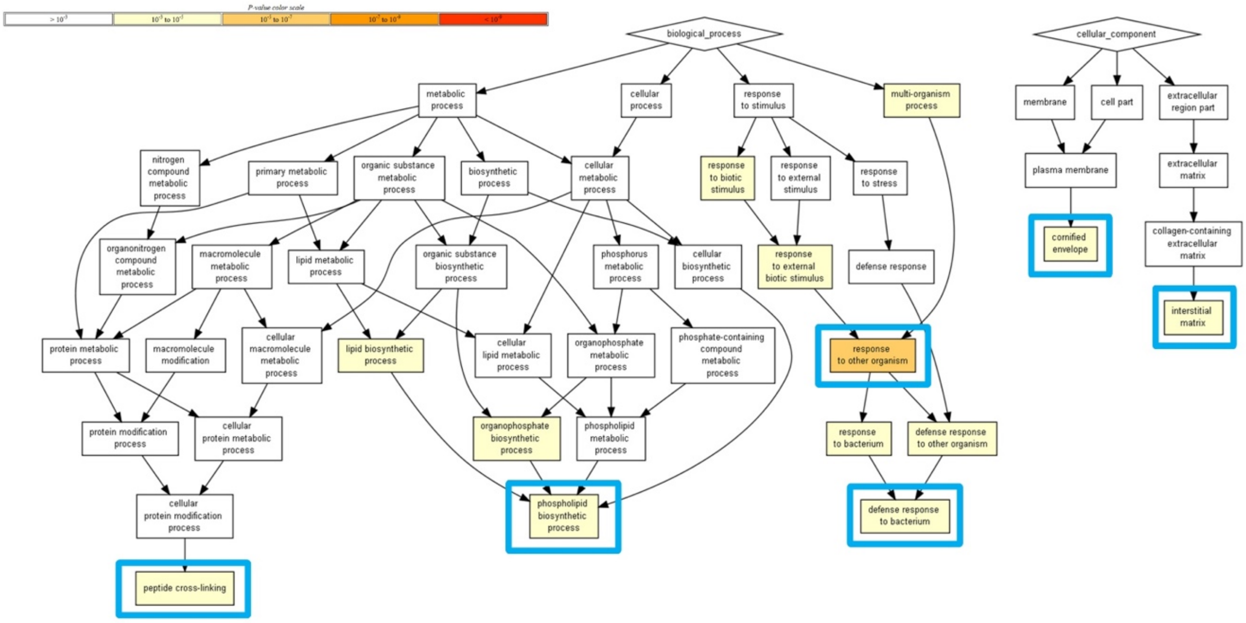

Biological process

GO term	Description	P-value	FDR q-value	Enrichment (N, B, n, b)
GO:0051707	response to other organism	7.22E-6	3.83E-2	1.90 (838,41,355,33)
GO:0042742	defense response to bacterium	3.73E-5	3.95E-2	2.39 (838,14,350,14)
GO:0018149	peptide cross-linking	7.47E-4	3.6E-1	8.66 (838,11,44,5)
GO:0008654	phospholipid biosynthetic process	3.24E-4	1.91E-1	7.51 (838,6,93,5)

Cellular component

GO term	Description	P-value	FDR q-value	Enrichment (N, B, n, b)
GO:0001533	cornified envelope	5E-6	3.46E-3	8.96 (838,17,44,8)
GO:0005614	interstitial matrix	3.47E-4	1.2E-1	79.81 (838,3,7,2)

Gorilla gene enrichment analysis was applied to RNA-seq data revealing mRNA changes in E18 alveolar bone of *Smoc2-/-*mutants. Significantly enriched GO terms genes (yellow color: *p*-value $<10^{-3}$; orange color: *p*-value $<10^{-5}$) include biological processes such as response to other organism, defense response to bacterium, peptide cross-linking, and phospholipid biosynthetic process. The cellular components altered due to Smoc2 deficiency are cornified envelope and interstitial matrix.

Figure S9

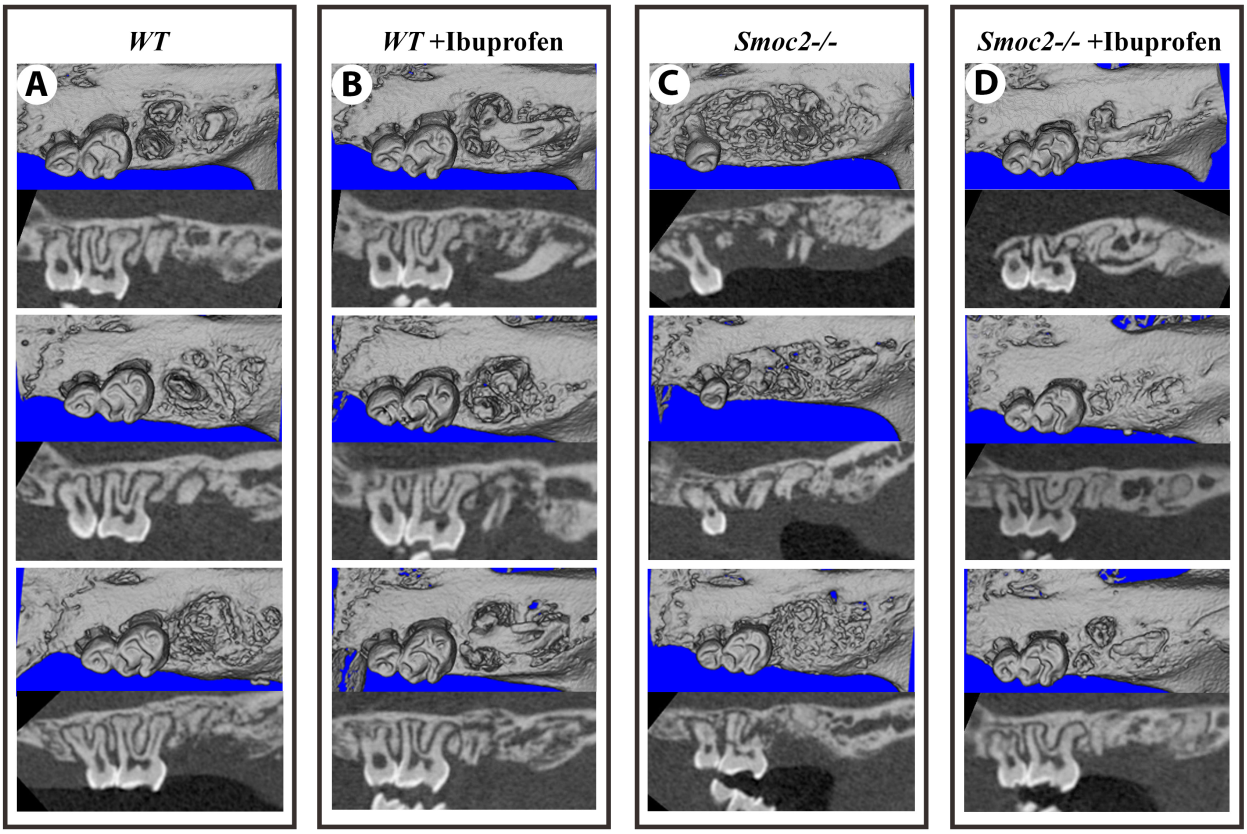

X-ray micro-computed tomography (μCT) images of the molars from two months-old *WT* (A,B) and *Smoc2-/-* (C,D) males, at 6-week post-injury, showing no differences between the non-ibuprofen-treated and ibuprofen-treated (10 days with ibuprofen provided in drinking water at a concentration of 30 mg/kg body weight) *WT* groups. Hence, no 2nd molar root resorption is ever observed post-injury in *WT* (A,B). *Smoc2-/-* mutants have destruction of bone and root around the injured first molar, a loss even extending into the 2nd molar roots (C). *Smoc2-/-* mutants after the same ibuprofen treatment (D) show no evidence of bone resorption, appearing similar to *WT*. However, at times a smaller 2nd molar root (suggesting resorption) is observed in 2 out of 7 *Smoc2-/-* mutants given ibuprofen. Image data are extracted from μCT analyzed samples using the Analyze 11.0 software, including the addition of a false blue background to 3-D images.

Figure S10

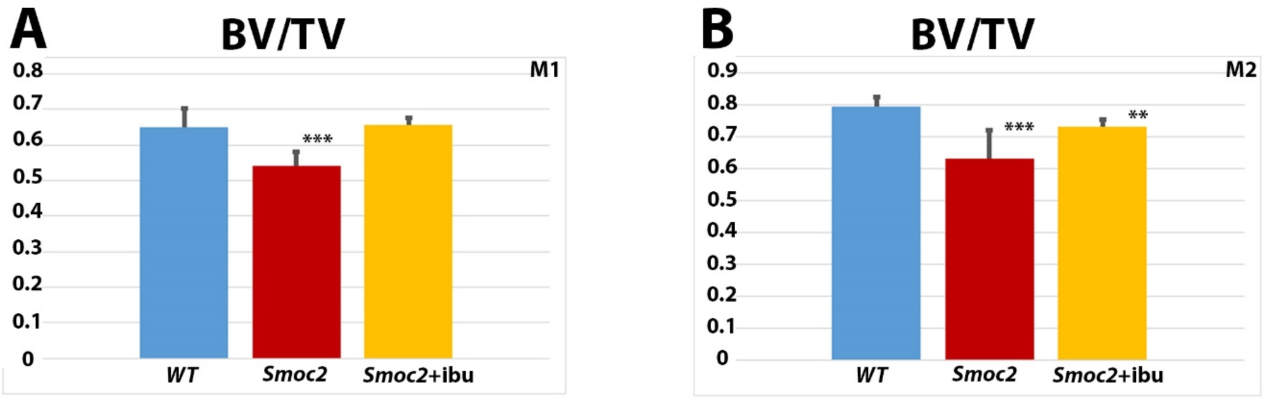

Six-week post-injury µCT data in which bone volume (BV) over total volume (TV) were calculated in the 1st (A) and 2nd (B) molar regions from two months-old *WT, Smoc2-/-* mutants, and *Smoc2-/-* mutants after ibuprofen treatment. *Smoc2-/-* mutants showed significantly reduced bone and root ossification volume. These defects were rescued in *Smoc2-/-* mutants treated for 10 days with ibuprofen (30 mg/kg). Data are presented as a BV/TV ratio (a value of 1 corresponding to 100% bone as assessed by µCT density calculation of ossification). This analysis was performed on 7 mice per group and Student's t-test was applied for statistical difference.

Figure S11

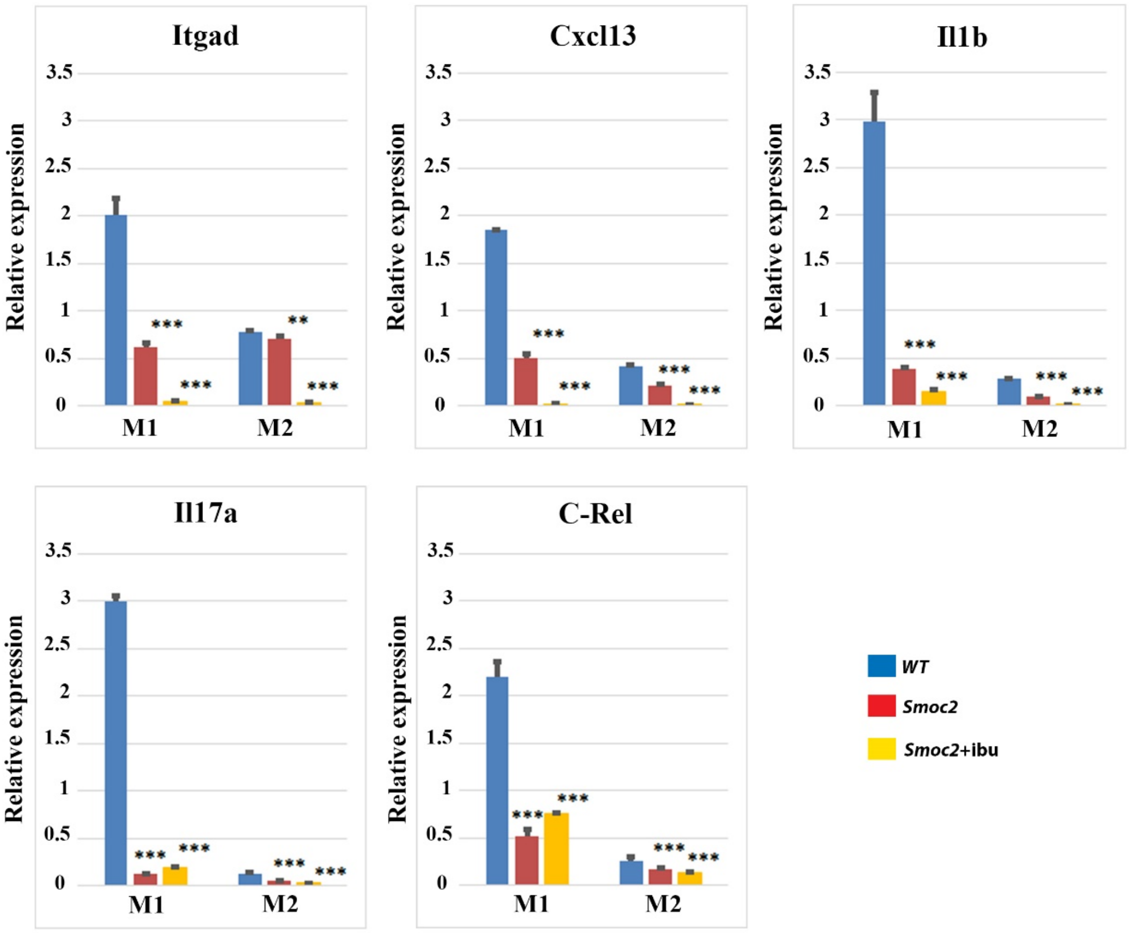

RT-PCR results from 7-day post-injury 1st and 2nd molar regions of *WT*, *Smoc2-/-* mutants, and *Smoc2-/-* mutants treated with ibuprofen (30 mg/kg). Six independent samples were analyzed for each group. Down-regulated targets shown here are *Itgad*, *Cxcl13*, *interleukin 1β* [*Il1b*], *Il17a*, and the NF-κB subunit *c-rel*.

Figure S12

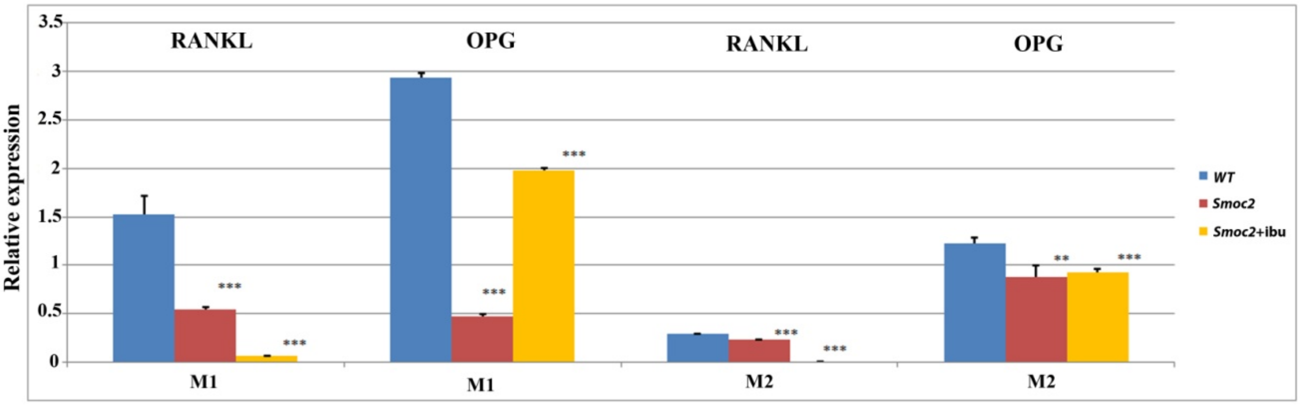

	RANKL/OPG	
	**M1**	**M2**
WT	0.52	0.23
Smoc2-/-	1.14	0.26
***Smoc2-/-* + Ibu**	0.03	0.01

RANKL/*OPG* ratio assessed by RT-PCR 7 days after surgery in bone underlying the 1st and 2nd molars in *WT*, *Smoc2-/-* mutants, and *Smoc2-/-* mutants treated with ibuprofen (30 mg/kg). Both *RANKL* and *OPG* are reduced in *Smoc2-/-* mutants. An increased bone remodeling response is indicated by an increased *RANKL*/*OPG* ratio in the first molar bone of *Smoc2-/-* mutants. This ratio is markedly reduced after ibuprofen treatment, with diminished *RANKL* in alveolar bone of both the 1st and 2nd molars. Seven independent samples were analyzed for each group.

Figure S13

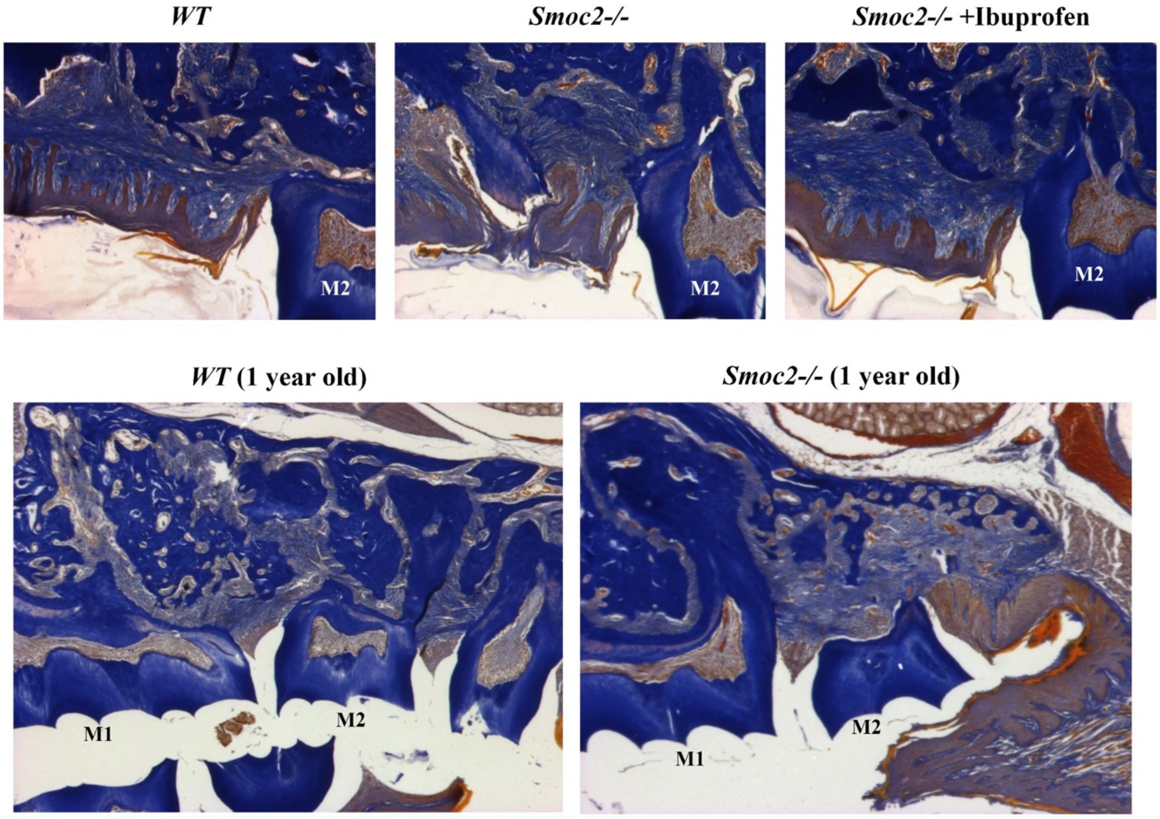

Mallory's trichrome stain on histological sections of 6-week post-injury *WT*, *Smoc2-/-* mutant, *Smoc2-/-* mutant following short-term ibuprofen treatment (10 days at 30 mg/kg), and 1-year-old *WT* and *Smoc2-/-* mutants reveals collagen deposition (intense blue staining). At the resorption area of the *Smoc2-/-* mutants and corresponding controls, no obvious changes in collagen staining was observed.

SUPPLEMENTAL METHODS

Alizarin red and alcian blue staining was performed after fixing embryos overnight in 95% ethanol. Then, coloring in 80 ml 95% ethanol + 20 ml acetic acid + 15 mg Alcian blue powder (8GX CI74240 SIGMA 25g A-3157) for approximately 48 hours for E15 and 72 hours for E18. After dehydrating in 95% ethanol for 2 days, samples were placed in 1% KOH in dH2O until bones were visible (approximately 6 hours). Finally, coloration in 15mg Alizarin red powder (CI58005 SIGMA 100g A-3757) diluted in 100 ml 1% KOH in dH2O overnight was followed by decoloration in 20% glycerol + 1% KOH in dH2O.

SUPPLEMENTAL TABLES

Table S1: Sequence of primers used for *Smoc2*, *Smoc2-GFP*, and sex genotyping.

Position	Sequence
Smoc2-Er	CTG TGC GAA GCT TTT CCA GGC TTG
Smoc2-Lf	CCA CTT TAT ACG GAA AGC TGA CCT G
Smoc2-Lr	GGC ATC ATT GCT GTT GTT GTG ATT GC
Smoc2-GFP-WT-r	ACC AGC AGC CCT TCA CAT AC
Smoc2-GFP-KI-f	GTT CGC ACA CCG GAT CTT
Smoc2-GFP-KI-r	GAA CTT CAG GGT CAG CTT GC
Sry-f	TTG TCT AGA GAG CAT GGA GGG CCA TGT CAA
Sry-r	CCA CTC CTC TGT GAC ACT TTA GCC CTC CGA

Table S2: Primer sequences used for real-time RT-PCR analysis.

NAME	Forward Primer	Reverse Primer
GFP	AAG TTC ATC TGC ACC ACC G	TCC TTG AAG AAG ATG GTG CG
Smoc2	CCC AAG CTC CCC TCA GAA G	GCC ACA CAC CTG GAC ACA T
Smoc1	CGG CCC CAG GTT CCT AAT AAG	GCA CTT GGC TCT CTG GTA CTC
Gapdh	AGG TCG TGT TGA ACG GAT TTG	TGT AGA CCA TGT AGT TGA GGT CA

Table S3: Prevalence of additional lower 4th molars in female and male *Smoc2-/-* mice.

Sex	Lower left molars	Lower right molars	Total number of *Smoc2-/-* mice	Prevalence in percentage
**Female**	5	5	40	25%
**Male**	3*	6*	43	16%

*Two male *Smoc2-/-* mice have additional 4th molars on both sides

Table S4: RNA-seq list from E14 molars: down-regulations in female *Smoc2-/-* embryos.

E14 Lower left molar down-regulation		
**Gene name**	**log2(Cmp1)**	**P-value (Cmp1)**
Smoc2	-3.199538631	1.3836E-152
Prap1	-2.731969466	1.45478E-05
Krt77	-2.601138443	6.5323E-05
Aqp8	-2.39258687	0.000258254
Cuzd1	-2.352254	0.00025779
Kcne2	-2.137588235	0.000663552
Pigr	-2.043490089	0.001858656
Tnfsf8	-1.94640338	4.76806E-05
Sult1d1	-1.904751814	0.001799265
Tmprss4	-1.898701662	0.001661714
Tpsg1	-1.876876099	0.000319064
Bglap	-1.871035208	1.98612E-06
Akr1c18	-1.695759562	0.003596391
Serpina1e	-1.67276569	0.010720047
Havcr1	-1.671871438	0.001454083
Mtnr1a	-1.63874683	0.006917907
Dmp1	-1.623399811	0.003309181
Guca2b	-1.603117863	0.003571115
Odam	-1.580032367	0.016057732
Sfrp4	-1.551244784	0.00010307
Akr1b7	-1.511497053	0.00696554
Cma1	-1.454970714	0.002340391
Enpp6	-1.448044796	0.000838038
H2-M2	-1.439099276	0.00900125
Zpbp2	-1.422636518	0.009239398
Prss34	-1.403785999	0.005737381
Tpsb2	-1.39399654	0.013900725
Slc25a34	-1.393149802	0.001035176
C3	-1.386985243	0.001220781
Sirpb1b	-1.364126393	0.036046334
Tdh	-1.357278968	0.03002097
Foxn1	-1.352739009	0.008837356

Platr17	-1.3419982	0.011423392
Sis	-1.327626951	0.037430357
Fcnaos	-1.32667227	0.038282964
Krtdap	-1.314952833	0.034561149
Wfdc17	-1.307317128	0.010899639
Vmn2r18	-1.292360995	0.048728663
Acpt	-1.286284004	0.029389801
Gzmk	-1.285301311	0.049676611
Adipoq	-1.275449633	0.039443402
Col10a1	-1.250794836	0.019190867
Zic2	-1.239428596	0.034878888
Rasgef1a	-1.235140458	0.002808926
Ccl22	-1.219558573	0.040437905
C4b	-1.214810142	0.000518501
Slamf7	-1.212007539	0.020944167
Oxct2a	-1.211590788	0.031132502
Mcpt2	-1.195425507	0.03109939
Wisp2	-1.194991434	0.013241595
Lsmem2	-1.19282472	0.011886323
BC021891	-1.190187911	2.3528E-05
Ripply1	-1.18764589	0.005691053
Ranbp3l	-1.147046889	0.001265142
Myh2	-1.144710394	0.008245954
Gal	-1.135636191	0.007134753
Mir6951	-1.135430115	0.039304789
Agbl1	-1.087416723	0.032749965
Clec3b/ Tetranectin	-1.087144659	0.045857146
Slc36a2	-1.078325855	0.019820373
Sectm1b Treg	-1.068823087	0.031077637
Pnpla1	-1.054213913	0.044701297
Krt75	-1.050661696	0.034917898
Mir3070a	-1.047973844	0.040909221
Tm4sf19	-1.045075113	0.001916879
Lipm	-1.044383463	0.025724378
Ly6g6e	-1.041186982	0.017418633
Ntn5	-1.038727476	0.000775008
Slc14a2	-1.031399531	0.017224756
Hrct1	-1.027252991	0.010491853
Ckmt2	-1.025126961	0.033107221
Efcab6	-1.023207828	0.020014435
Cpa3	-1.010967643	0.01176532
Lrrc14b	-1.00424848	0.002751275
Mcpt4	-1.003722819	0.03324313

Table S5: RNA-seq list from E14 molars: up-regulations in female *Smoc2-/-* embryos.

**E14 Lower left molar up-regulation**		
**Gene name**	**log2(Cmp1)**	**P-value (Cmp1)**
Afp	2.745389551	2.8307E-05
Apob	2.210807245	0.000438456
Fga	2.095581493	0.001115386
Cnpy1	1.968752237	0.002719378
Apoa4	1.765600751	0.006454816
Hoxd1	1.695735215	0.000295882
Gphb5	1.669335427	0.010798346
Kng1	1.666622962	0.007583643
Haglr	1.580367656	0.000851453
Wfdc18	1.551034093	0.008701363
Rfx4	1.483482549	7.18658E-05
Nlrp2	1.480901161	0.002403456
Fkbp6	1.461586655	0.023055129
Wfdc21	1.459194483	0.015581156
Apoa1	1.444144326	0.010188418
Sun3	1.42575898	0.021019442
Slc39a12	1.417806787	0.001091862
Mir7091	1.383789242	0.031724533
Slc22a8	1.354955316	0.038591256
Olfr876	1.330549728	0.031215434
Spink1	1.325087501	0.024262133
Ccnd3-ps	1.298813197	0.044556667
Nr2e1	1.287107155	0.047559045
Casp16-ps	1.211079522	0.037319809
Dynap	1.195355337	0.046187759
Fgb	1.187964157	0.020557017
Cyp3a13	1.186437	0.043379567
Rhag	1.174323027	0.045374135
Hmx1	1.160675569	0.020448925
Diras1	1.148693712	0.017697455
Serpina1b	1.146376761	0.039411302
Sox3	1.143906785	0.002861372
Eqtn	1.139507311	0.018880621
Slc6a1	1.118254044	0.015103081
Foxb1	1.07402558	0.04733504

Table S6: RNA-seq list from E18 alveolar bone: down-regulations in female *Smoc2-/-* embryos.

E18 Lower left bone down-regulation		
**Gene name**	**log2(Cmp2)**	**P-value (Cmp2)**
Smoc2	-3.526311044	3.3471E-183
Tpi-rs11	-2.180319065	0.000603923
Gpr22	-1.796381177	0.001673414
Fmr1nb	-1.780737025	0.006481526
Xlr3b	-1.721365763	0.000319849
Odf3b	-1.630861979	0.005286463
Ppy	-1.619496865	0.006511067
Dspp	-1.602806927	0.014328382
Bpifa1	-1.581895892	0.015784269
Gh	-1.573238153	0.01459984
Treml1	-1.569628431	0.000977314
Itgad	-1.563323652	0.012501519
Hist1h3c	-1.5602441	0.007970537
Mannr	-1.518344001	0.017591371
Abca14	-1.49800235	0.020526845
March4	-1.459697911	0.014862706
Neurod2	-1.443011889	0.027699984
Zic4	-1.442772063	0.017637193
Ankub1	-1.440983192	0.027633356
Prss56	-1.40818783	0.026282487
Scrt1	-1.399313196	0.008708598
Pak7	-1.392068128	1.87828E-05
Cym	-1.382662022	0.024677409
Actl6b	-1.366336908	0.009043513
Hoxa9	-1.357500084	0.038626392
Tecta	-1.345495724	0.001739596
Tmprss6	-1.339376744	0.036130082
Cxcl13	-1.33530037	0.004137394
Tekt4	-1.288328201	0.049664103
Smkr-ps	-1.284332316	0.023562586
Mir673	-1.278578119	0.033241221
Cdk5r2	-1.276883362	0.006777658
Dscaml1	-1.270673259	0.001122716
Xlr4b	-1.266026654	0.017148516
Il1rapl1	-1.206774891	0.007216544
Chga	-1.192831964	0.008998984
Myt1l	-1.183896408	0.013017563
Csn2	-1.166197697	0.030608235
Syt4	-1.157134742	0.002331657
Rmst	-1.13315062	0.037475522
Mrgprb13	-1.119478849	0.025591044
Atp8b3	-1.108131622	0.016511037
Mir5110	-1.107840855	0.024886482
Timd4	-1.101266117	0.01442262

Igsf21	-1.072035188	0.02662931
Mast1	-1.063738906	0.034946131
Epyc	-1.015714326	0.041096859

Table S7: RNA-seq list from E18 alveolar bone: up-regulations in female *Smoc2-/-* embryos.

E18 Lower left bone up-regulation		
**Gene name**	**log2(Cmp2)**	**P-value (Cmp2)**
Klk14	3.159685979	2.23435E-07
Gsdmc	3.147797198	2.04271E-12
Teddm3	2.991884627	2.39223E-07
Wfdc5	2.90071236	7.4646E-06
Alox12b	2.868620842	7.75272E-07
Tgm3	2.850688723	3.59591E-07
Rptn	2.781491608	4.08564E-06
Sprr3	2.65980732	1.31574E-05
Ly6g6c	2.598389936	5.62034E-05
Krt4	2.580887845	3.88581E-05
Prss27	2.571848436	4.64194E-05
Klk12	2.571725119	8.39219E-05
Ly6d	2.532295082	3.70889E-05
Idi2	2.465012375	1.40128E-05
Lce3b	2.457626853	0.000128343
Slurp1	2.387164877	0.000114222
Serpinb3a	2.366554219	0.000272107
Lce3e	2.332259768	7.10752E-05
Csta1	2.254406065	1.07478E-05
Krt78	2.249673619	0.000200931
Kcnk7	2.22809819	0.000129765
Slc25a48	2.218461396	2.74944E-07
Krt13	2.158219167	0.00094526
Klk13	2.144809264	1.43752E-05
Cnfn	2.126472203	0.00114772
Defb14	2.124494442	0.001140504
Lypd5	2.11409192	0.000426466
Cyp2f2	2.083653263	0.000422642
Paqr5	2.078503132	1.05693E-05
Aldh3b2	2.046810948	2.40417E-06
Dsg3	2.044610981	0.000298158
Irx4	2.036160134	0.000592921
Gjb2	2.030671366	2.92921E-05
Lor	2.029458702	0.000143404
Them5	2.021483074	0.000238702
Trex2	2.006843435	0.001886382
Slc28a3	1.996966059	0.000853127
Psapl1	1.989460002	0.00192624

Lypd3	1.988896477	0.000289672
Crisp1	1.966663167	0.002657897
Pinlyp	1.964595819	0.002713012
Lce3a	1.963343334	0.000302389
Klk7	1.943095134	0.002793737
Krt16	1.930154915	0.00206791
Spink5	1.92266907	0.002370482
Krtdap	1.910989904	0.002110819
Serpinb12	1.910964627	0.00178339
H60c	1.899968999	0.000176454
Krt6a	1.870938412	0.000495535
Krt15	1.822489622	0.001632884
Lce3c	1.817032026	0.003812285
Dmkn	1.807227842	0.005275779
Calml3	1.800207116	0.000888906
Sptlc3	1.771029763	0.000450942
Kprp	1.760557803	0.002189338
Clic3	1.750527855	0.00059869
Fgfbp1	1.739470308	0.00104318
Lce3f	1.722644584	0.002703273
Alox12e	1.677616991	0.001336073
Defb4	1.676600205	0.010388693
Fam83a	1.646281913	0.001929835
Nkpd1	1.616236557	0.005522935
Gjb6	1.609585387	0.001438882
Cysrt1	1.603402675	0.00152511
Fmo9	1.598401702	0.014870287
S100a14	1.591931118	0.000584604
Fam26d	1.58370128	0.010077756
Fam25c	1.579952486	0.01620246
Vtcn1	1.579923048	0.008826642
Klk9	1.570656283	0.002967533
Bnipl	1.564620327	0.00018229
Acer1	1.558084746	0.017614736
Slc5a9	1.555968916	0.004940862
Upb1	1.549342578	0.000177474
Cyp2w1	1.542604447	0.011980559
Flg2	1.531525814	0.006198143
Ucn2	1.529333595	0.000309248
Aim1l	1.519347166	3.39493E-05
Ephx3	1.517184383	0.001757042
Calm4	1.51275745	0.01155509
Pdzk1ip1	1.51068788	0.001097093
Crct1	1.506354693	0.005984302
Krt6b	1.504388174	0.021918387
Mir7013	1.501532547	0.020830613
Nr1h5	1.496186423	0.016823158
Prodh2	1.490773232	0.022446278

Platr16	1.482812649	0.02366305
Asprv1	1.479714242	0.003320927
Rps8-ps1	1.478005794	0.023444168
Tmprss11b	1.472666546	0.003512137
Tmem211	1.467906057	0.022552851
Sdr9c7	1.46278547	0.023575981
Lipk	1.460990464	0.025747186
Nccrp1	1.46093315	0.004948222
Capns2	1.459599167	0.001807537
Rbbp8nl	1.457721812	0.002288432
Slc38a5	1.451974044	0.010856928
Pla2g2f	1.450078566	0.024028478
Eif4a-ps4	1.448786117	0.026026727
Fam135b	1.441134408	0.004347333
Hrnr	1.440368215	0.026311642
Gjb4	1.439789896	0.024954125
Fam83c	1.438803342	0.00522369
Rbp2	1.437432894	0.003297191
Ccdc182	1.431499316	0.026636702
Serpina3h	1.430261878	0.029079692
Rhbg	1.429543288	0.009351971
Cyp4f39	1.415701164	0.023466628
Olfr1456-ps1	1.41293359	0.026191119
Foxe1	1.412836553	0.004926926
Padi1	1.406782486	0.001185506
Serpinb5	1.406576336	0.003470413
Lrrc52	1.406299604	0.024605445
Mir337	1.396432169	0.026836784
Ces1h	1.394630371	0.023153097
Fat2	1.393785045	0.008368591
Ptk6	1.390866449	0.027789942
Fam92b	1.385641268	0.032775175
Sprr1a	1.3824493	0.02557846
Il1f6	1.379049141	0.02092159
Cyp2a5	1.375773461	0.0169132
Sytl1	1.361049553	0.00039099
Oaz3	1.35758871	0.029179279
Npbwr1	1.356950071	0.034449299
Sprr1b	1.341095087	0.028521007
Mst1	1.340803846	0.009510315
Nox1	1.328934347	0.034529612
Caps2	1.325086608	0.02226272
Cyp2t4	1.323157861	0.042957562
Fam166a	1.322729637	0.043377405
C6	1.322550739	0.026527814
Pla2g4f	1.320010386	0.027502632
Klk5	1.31721307	0.039746864
Sh2d1a	1.315296792	0.043829509

Krt31	1.312335557	0.037442873
Cyp3a13	1.305183198	0.025073409
Ttc22	1.301337003	0.005152229
Pkd1l3	1.30056626	0.003266025
Cers3	1.297150557	0.00077824
Tmprss11d	1.294947319	0.038058755
Chn1os1	1.293037822	0.044434668
Lce1e	1.288805347	0.026204369
Aloxe3	1.28432733	0.000973003
Cldn14	1.282207036	0.016143159
Prl8a6	1.282004489	0.033523367
Pkp1	1.281100095	0.009947712
Hemgn	1.270180615	0.03090284
Krt23	1.268328277	0.03173657
Lgals7	1.264533303	0.012218814
Tgtp1	1.261642586	0.047279744
Sult6b2	1.260494472	0.047884464
Pydc4	1.255866332	0.011974516
Tns4	1.253961362	0.000476521
Wnt3	1.253783005	0.010269818
Ly6g6e	1.252154235	0.006960722
Slco1b2	1.246852242	0.036385351
Ube2u	1.243407736	0.027337852
Aqp3	1.241470851	0.00171669
Myoc	1.235896247	0.012350358
Krt1	1.235397053	0.04278664
Krt5	1.233232012	0.022575913
Scnn1g	1.232133174	0.005854265
mt-Tw	1.230253746	0.002419357
Pla2g2c	1.223880385	0.049140774
Serpinb10	1.216216377	0.042262309
Mir8091	1.212588144	0.008647073
Psors1c2	1.205439775	0.008497058
Htr3a	1.200054886	0.000291178
Ppl	1.189725764	0.009588732
Mageb3	1.187610746	0.031915883
Sel1l2	1.171187257	0.049597203
Spib	1.161224936	0.000389357
Rassf6	1.160977314	0.027290053
Dsc2	1.143369024	0.015615605
Aox4	1.139012838	0.003055068
Fxyd3	1.120428567	0.014219153
Tfap2b	1.119960916	0.000461796
Ace2	1.116871816	0.024778368
Rpl23a-ps5	1.112451002	0.047159427
Ccdc151	1.111493446	0.001642669
Sox15	1.110829708	0.021062475
S1pr5	1.11035656	0.031178351

Arg1	1.106177763	0.006231969
Zfa-ps	1.092247065	0.049195529
Sbsn	1.089327606	1.38941E-06
Cst6	1.089319387	0.041160998
Trim29	1.087600714	0.043332147
Upk3bl	1.087541281	0.001042759
Usp18	1.087360454	0.003722234
Slc6a14	1.086472912	0.040822808
Dbx2	1.082166383	0.001005794
Etnk2	1.081889048	0.001066552
Sult2b1	1.081195115	0.016527008
Gjb3	1.071608461	0.011356224
Ces2g	1.068019036	0.002877803
Tnk1	1.067129677	0.02485976
Treh	1.06514264	0.044135672
Ankrd22	1.049324981	0.022537094
Cldn23	1.040997522	0.023882855
Lad1	1.039626241	0.037463491
Irx2	1.029484952	0.026067576
G6b	1.028390417	0.041976977
Edar	1.01737674	0.003901827
Endou	1.016082232	0.024520202
Pof1b	1.008750207	0.031192179
Ccdc144b	1.003600986	0.00761951

Table S8: Primer sequences used for real-time RT-PCR analysis on inflammation.

NAME	Forward Primer	Reverse Primer
aSMA	GGA CGT ACA ACT GGT ATT GTG C	TCG GCA GTA GTC ACG AAG GA
Cox2	TGC ACT ATG GTT ACA AAA GCT GG	TCA GGA AGC TCC TTA TTT CCC TT
C-rel	AGA GGG GAA TGC GGT TTA GAT	TTC TGG TCC AAA TTC TGC TTC AT
Cxcl13	ATA TGT GTG AAT CCT CGT GCC A	GGG AGT TGA AGA CAG ACT TTT GC
CyclinD1	GCG TAC CCT GAC ACC AAT CTC	CTC CTC TTC GCA CTT CTG CTC
Egf	AGC ATC TCT CGG ATT GAC CCA	CCT GTC CCG TTA AGG AAA ACT CT
Ifn-g	ATG AAC GCT ACA CAC TGC ATC	CCA TCC TTT TGC CAG TTC CTC
Il-1b	GAA ATG CCA CCT TTT GAC AGT G	TGG ATG CTC TCA TCA GGA CAG
Il-17a	TTT AAC TCC CTT GGC GCA AAA	CTT TCC CTC CGC ATT GAC AC

Il-17b	GAG TAA AGC CCT ACG CTC GAA	CTC CTC TTG TTG GAC AAC CAC
Il-36a	ATG CCA ATA TCT GGA CAC TCT TG	AGA GAG GCT TTT ACA GGT TCC TT
Il-6	CTG CAA GAG ACT TCC ATC CAG	AGT GGT ATA GAC AGG TCT GTT GG
iNOS	ACA TCG ACC CGT CCA CAG TAT	CAG AGG GGT AGG CTT GTC TC
Itgad	TCC TGC GCC TTA ACT TAT CCC	GCT CCT GCT TAC AGT TCT TCT C
M-CSF	ATG AGC AGG AGT ATT GCC AAG G	TCC ATT CCC AAT CAT GTG GCT A
Mmp9	GCA GAG GCA TAC TTG TAC CG	TGA TGT TAT GAT GGT CCC ACT TG
OPG	CCT TGC CCT GAC CAC TCT TAT	CAC ACA CTC GGT TGT GGG T
RANKL	CAG CAT CGC TCT GTT CCT GTA	CTG CGT TTT CAT GGA GTC TCA
Tgfb1	CTT CAA TAC GTC AGA CAT TCG GG	GTA ACG CCA GGA ATT GTT GCT A
Tgfb2	TCG ACA TGG ATC AGT TTA TGC G	CCC TGG TAC TGT TGT AGA TGG A
Tnfa	CCT GTA GCC CAC GTC GTA G	GGG AGT AGA CAA GGT ACA ACC C
Vegfa	GCA CAT AGA GAG AAT GAG CTT CC	CTC CGC TCT GAA CAA GGC T
Gapdh	AGG TCG TGT TGA ACG GAT TTG	TGT AGA CCA TGT AGT TGA GGT CA

Table S9: Prevalence of *Smoc2-/-* 1 year old mice with alveolar bone destruction.

**Sex**	**Number of *Smoc2-/-* 1 year old mice with alveolar bone destruction**	**Total number of *Smoc2-/-* 1 year old mice**	**Prevalence in percentage**
**Female**	3	5	60%
**Male**	1	5	20%
